# Impact, economic evaluation, and sustainability of integrated vector management in urban settings to prevent vector-borne diseases: a scoping review

**DOI:** 10.1186/s40249-018-0464-x

**Published:** 2018-09-03

**Authors:** Jorge Marcos-Marcos, Antonio Olry de Labry-Lima, Silvia Toro-Cardenas, Marina Lacasaña, Stéphanie Degroote, Valéry Ridde, Clara Bermudez-Tamayo

**Affiliations:** 1 Public Health Research Group, University of Alicante, Alicante, Spain; 2 Andalusian School of Public Health, Granada, Spain; 3 Biomedical Research Centre Network for Epidemiology and Public Health (CIBERESP), Madrid, Spain; 4 Biomedical Research Centre (ibs.GRANADA), Granada, Spain; 5 Public Health Research Institute (IRSPUM), University of Montreal, Montreal, Quebec Canada; 6 IRD (French Institute for Research on Sustainable Development), CEPED (IRD-Université Paris Descartes), Universités Paris Sorbonne Cités, ERL INSERM SAGESUD, Paris, France

**Keywords:** Vector-borne diseases, Integrated vector management, Urban health, Intervention, Scoping review

## Abstract

**Background:**

The control of vector-borne diseases (VBD) is one of the greatest challenges on the global health agenda. Rapid and uncontrolled urbanization has heightened the interest in addressing these challenges through an integrated vector management (IVM) approach. The aim was to identify components related to impacts, economic evaluation, and sustainability that might contribute to this integrated approach to VBD prevention.

**Main body:**

We conducted a scoping review of available literature (2000–2016) using PubMed, Web of Science, Cochrane, CINAHL, Econlit, LILACS, Global Health Database, Scopus, and Embase, as well as Tropical Diseases Bulletin, WHOLIS, WHO Pesticide Evaluation Scheme, and Google Scholar. MeSH terms and free-text terms were used. A data extraction form was used, including TIDieR and ASTAIRE. MMAT and CHEERS were used to evaluate quality.

Of the 42 documents reviewed, 30 were focused on dengue, eight on malaria, and two on leishmaniasis. More than a half of the studies were conducted in the Americas. Half used a quantitative descriptive approach (*n* = 21), followed by cluster randomized controlled trials (*n* = 11). Regarding impacts, outcomes were: a) use of measures for vector control; b) vector control; c) health measures; and d) social measures. IVM reduced breeding sites, the entomology index, and parasite rates. Results were heterogeneous, with variable magnitudes, but in all cases were favourable to the intervention. Evidence of IVM impacts on health outcomes was very limited but showed reduced incidence. Social outcomes were improved abilities and capacities, empowerment, and community knowledge. Regarding economic evaluation, only four studies performed an economic analysis, and intervention benefits outweighed costs. Cost-effectiveness was dependent on illness incidence. The results provided key elements to analyze sustainability in terms of three dimensions (social, economic, and environmental), emphasizing the implementation of a community-focused eco-bio-social approach.

**Conclusions:**

IVM has an impact on reducing vector breeding sites and the entomology index, but evidence of impacts on health outcomes is limited. Social outcomes are improved abilities and capacities, empowerment, and community knowledge. Economic evaluations are scarce, and cost-effectiveness is dependent on illness incidence. Community capacity building is the main component of sustainability, together with collaboration, institutionalization, and routinization of activities. Findings indicate a great heterogeneity in the interventions and highlight the need for characterizing interventions rigorously to facilitate transferability.

**Electronic supplementary material:**

The online version of this article (10.1186/s40249-018-0464-x) contains supplementary material, which is available to authorized users.

## Multilingual abstracts

Please see Additional file 1 for translations of the abstract into the five official working languages of the United Nations.

## Background

Integrated vector management (IVM) is an approach using both chemical and non-chemical methods, including environmental management [1, 2]. It is part of a comprehensive strategy encompassing a variety of other vector control methods, such as collaboration with the health sector and other sectors, educational campaigns, advocacy, social mobilization, evidence-based decision making, and capacity building [3]. IVM may also refer to the concurrent control of multiple diseases transmitted by different vectors in a given zone, or to one tool controlling several vector-borne diseases transmitted by the same vector [4]. This strategic framework, adopted in 2004 for all vector-borne diseases (VBDs), is considered a rational decision-making approach for the optimal use of resources for vector control [5].

The financial crisis of recent years has raised doubts about the possibility of continuing the development of current interventions [6], which in turn has increased vulnerability factors in many populations [7]. This period of crisis has also consequently reinforced interest in knowing about and fostering the impacts, cost-effectiveness, and sustainability of programs and interventions. As part of the plan to improve the epidemiological situation, World Health Organization (WHO) began, in 2004, to concentrate on developing IVM [5]. However, as implementing integrated approaches is a slow and complex process, IVM remains an approach with great promise for the control of vector-borne and other infectious diseases related to poverty worldwide [8].

Among key vector control elements, the scientific literature highlights program administration, vector surveillance, control activities, public education, and intergovernmental coordination [2, 9]. In relation to implementation, policy-setting, capacity-building and advocacy, the decision-making process is essential to IVM [10]. Our aim in this paper is to contribute to the understanding of key factors that can positively influence population health, by considering the management of available human and financial resources. Specifically, the aim of this review is to identify components related to impact, cost-effectiveness, and sustainability that may facilitate implementation of an IVM approach in urban settings to prevent vector-borne diseases.

## Methods

### Scoping topic definition

We used an eDelphi survey to identify the six topics considered highest priority by a panel of 109 international experts (43% research sector, 52% public health sector, 5% private sector). The eDelphi was a three-round process: 1) participants suggested topics to be considered, resulting in more than 80 being proposed; 2) the proposed topics were rated from “1–eliminate” to “5–top priority”; 3) the 20 subjects rated 4 or 5 by more than 65% of the participants were then rated a second time. By the end of the process, the present topic had obtained the mean score of 4.08 ± 0.71 and was ranked the 3rd (rated 4 or 5 by 79% of participants in the final round).

### Search strategy

We reviewed the available literature using electronic databases: PubMed, Web of Science Database, Cochrane Library, CINAHL Complete Database, Econlit, LILACS, Global Health Database (CABS abstracts + Public Health and Tropical Medicine), Scopus, and Embase. This was complemented with a search of the following resources: Tropical Diseases Bulletin, WHO Pesticide Evaluation Scheme WHOLIS, and Google Scholar.

Key words included: program evaluation, cost analysis, impact analysis, cost-effectiveness, sustainability, vector-borne diseases, integrated vector management, urban areas, and their alternative expressions. Both MeSH (Medical Subject Headings) terms and free-text terms were used. The search strategy adopted for the different databases was validated by a librarian specialized in public health and is described in Additional file 2.

### Inclusion criteria

The included studies: 1) examined programs or interventions addressed by an IVM program; 2) referred to VBDs included in the WHO list; 3) presented relevant outcome measures in relation to impact, economic evaluation, and/or sustainability; 4) were conducted in urban settings according to 2014 United Nations criteria [11]; 5) were written in English, Spanish, French, or Portuguese; and 5) were published between 2000 and 2016.

### Operational definitions

*Integrated vector management* involves both chemical and non-chemical methods, including environmental management [5]. It is a comprehensive strategy characterized by WHO [10] as integrating vector control methods, educational campaigns**,** collaboration with the health sector and other sectors, advocacy, social mobilization**,** evidence-based decision-making, and/or capacity-building [2, 10, 12].

*Impact* refers to the extent to which a given intervention or service produces health outcomes in the individuals to whom it is offered [13]. It can be also evaluated in relation to various objectives, such as meeting societal needs [14]. Impact reflects the effects of an action or intervention [15].

*Economic evaluation* is a comparative analysis of alternative courses of action in terms of both their costs and consequences [16].

*Sustainability* is a condition for ensuring programs can continue to operate over the long term. It can be mainly connected with using, over an extended period of time, the components and activities needed to achieve outcomes that will control the illness [17].

### Data extraction and analysis

References were saved in a Zotero library and reviewed to identify potentially relevant papers. Titles and abstracts were assessed independently by two reviewers to determine whether papers met the inclusion criteria; those satisfying the criteria were saved as potential documents (first screening). Additional sources were obtained after screening by cross-checking the references of previously identified papers. Differences between reviewers were resolved by consensus by a third reviewer. The selected documents were then assessed with full text screening independently by two reviewers (second screening).

The documents were subjected to an evaluation of their methodologies using the Mixed Method Appraisal Tool (MMAT) [18] and, for economic evaluation studies, the Consolidated Health Economic Evaluation Reporting Standards (CHEERS) [19].

Information was extracted from each document using an Excel form to capture general information and two different tools, Template for Intervention Description and Replication (TIDieR) [20] and AnalySe de la Transférabilité et Accompagnement à l’adaptation des InteRventions en promotion de la santE (ASTAIRE) [21], to analyze the potential for transferability of the interventions. Specific information, such as intervention type and outcomes measured, was also included. Each item was classified as either reported or not fully reported (including omitted or poorly reported).

Data were synthesized and content analysis was performed according to categories related to the three dimensions previously established: impact evaluation, economic assessment, and sustainability. This facilitated comparison among the different studies and identification of gaps in public health policy and research in accordance with the IVM.

## Results

**Fig. 1:**
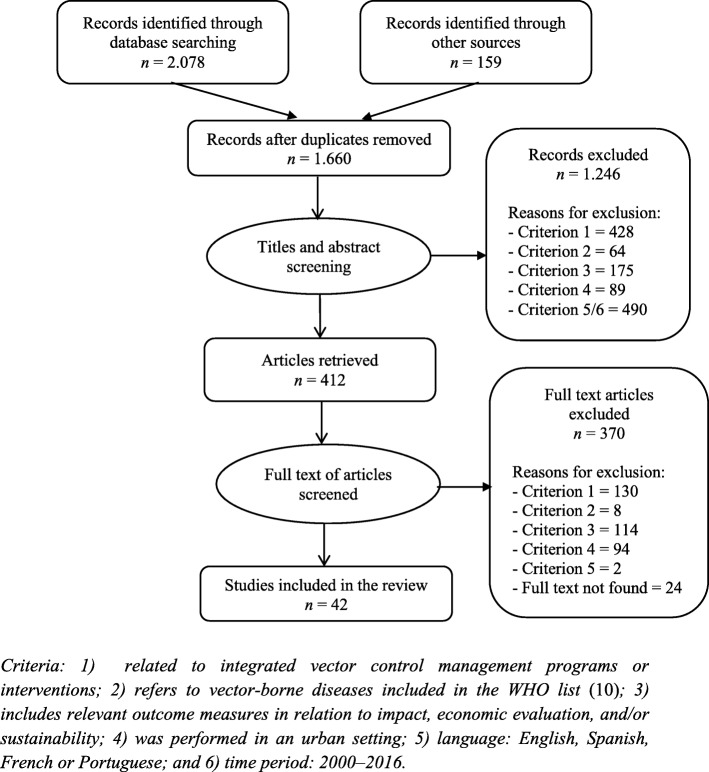
Studies selection process

A total of 1660 documents were retrieved, of which 409 were potentially eligible and 42 were included in the review. Figure 1 presents a flow diagram of the studies selection process. Additional file 3 lists the selected studies.

### Description of studies included

The studies reviewed had worldwide distribution. More than half were conducted in the Americas region (58.1%, *n* = 25), including Colombia, Cuba, Brazil, Argentina, Ecuador, Mexico, Uruguay, Venezuela, Brazil, and the United States, followed by the regions of Asia (18.6%), Africa (18.6%), and Oceania (4.7%) (Table 1). Of the 42 documents reviewed, 30 (71.4%) were focused on dengue vectors, eight (19%) on malaria vectors, and two on leishmaniasis. Half of the studies used a quantitative descriptive approach (*n* = 21); the others used quantitative randomized controlled trials (cluster) (*n* = 11), quantitative non-randomized methods (*n* = 6), and mixed methods (*n* = 4).

**Table 1:** Characteristics of studies

Characteristics	*n* (%)
Number of studies reviewed	42
Region^a^	
Americas	25 (58.1)
Africa	8 (18.6)
Asia	8 (18.6)
Oceania	2 (4.7)
Disease concerned	
Dengue	30 (71.4)
Malaria	8 (19.0)
Leishmaniasis	2 (4.8)
Several	2 (4.8)
Study type	
Quantitative descriptive	21 (50.0)
Quantitative randomized controlled (trials)	11 (26.2)
Mixed methods	4 (9.5)
Quantitative non-randomized	6 (14.3)
Intervention Type	
Vertical approach	
Educational intervention (EI) for vector control [22–31] and intersectoral activities	22 (52.4)
EI and road infrastructure modifications with use of slow-release insecticides [32]	
EI with an entomological survey [33]	
EI with periodic visits to houses [34]	
EI with treatment with larvicides, combined with insecticide spraying [35–39]	
EI and spraying with activities to control immature mosquitoes [40]	
Indoor ultra-low volume (ULV) application and EI [41]	
Large-scale installation of insecticide-treated screens and curtains and EI [42]	
Long-lasting insecticide-treated curtains, water container covers, and EI [43, 38]	
Community-based program	
Community-based control [44–48]	20 (47.6)
Ecosystem and environmental community-based approach [49–53]	
Community-based larviciding program [54–58]	
Community-based approach with window screening, ceilings, and closed eaves [59]	
Community-based intervention with entomological surveillance of vector [60]	
Environmental management, high-resolution aerial photography with ground-based validation [61]	
Mass control of vector in street catch basins and community participation [62]	
Support to program managers with situational information and community involvement [63]	

^a^ Some studies were conducted in several countries

### Methods used

**Fig. 2:**
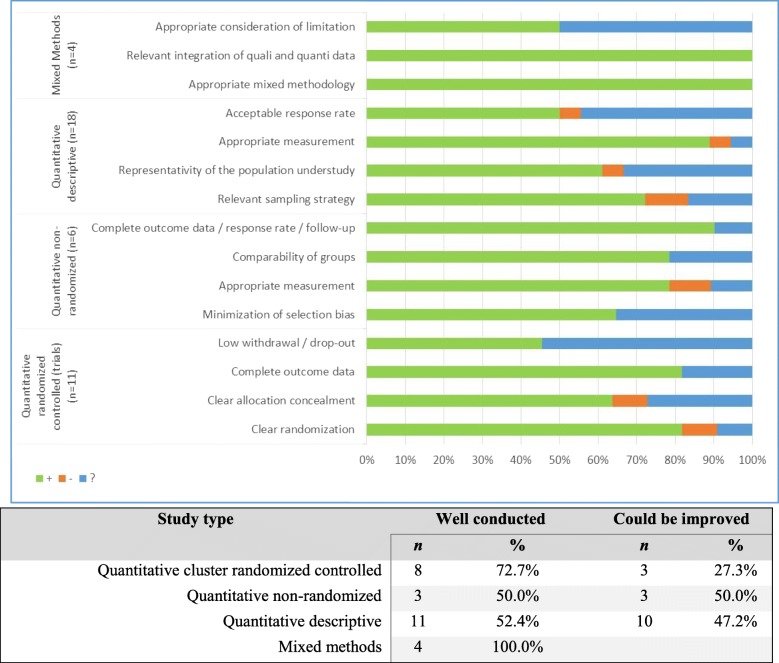
Quality of studies according to Mixed Method Appraisal Tool

Figure 2 shows the results produced by the MMAT analysis [18]: 72.7% of the quantitative randomized studies were considered well conducted (three or more items addressed), as were half of the quantitative non-randomized studies, 52.4% of the quantitative descriptive studies, and all the mixed methods studies.

### Descriptions of interventions

The studies analyzed 22 vertical-approach and 20 community-approach interventions.

Vertical-approach interventions took a variety of forms: a) educational interventions (EIs) for vector control within the community plus intersectoral work [22–31]; b) EIs plus modification of road infrastructure with use of slow-release insecticide [32]; c) EIs supported by an entomological survey [33]; d) EIs with periodic visits to houses for follow-up [34]; f) EIs plus larvicide treatment and insecticide spraying [35–39]; g) EIs plus spraying, with activities to control immature mosquitoes [40]; h) indoor ultra-low volume (ULV) application [41]; i) EI plus large-scale installation of insecticide-treated screens and curtains [42]; and j) EI plus long-lasting insecticide-treated curtains and water container covers [43, 38] (Table 1).

Other groups of interventions (*n* = 20) involved a community approach with various components [44–63]: 1) community-based control [44–48]; 2) ecosystemic and environmental community-based approaches [49–53]; 3) community-based larviciding programs [54–58]; 4) community-based approach with window screens, ceilings, and closed eaves [59]; 5) community-based intervention with entomological vector surveillance [60]; 6) environmental management and high-resolution aerial photography with ground-based validation [61]; 7) mass control of vectors in street catch basins and community participation [62]; and 8) support to program managers with situational information and community involvement [63].

**Fig. 3:**
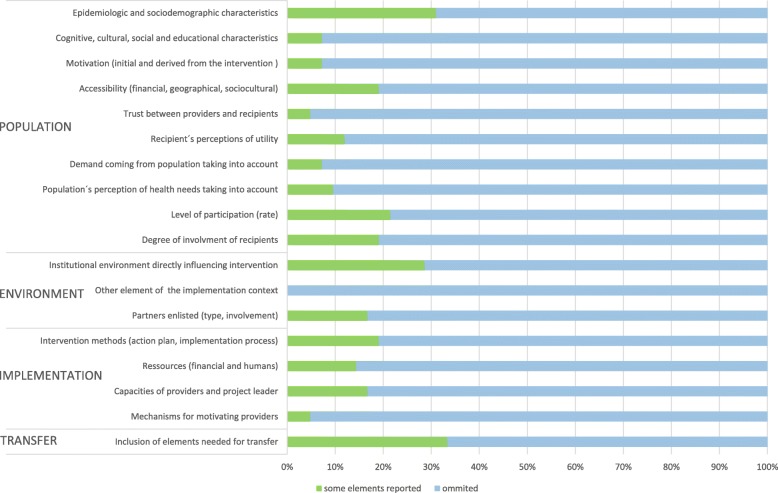
Description of interventions according to AnalySe de la Transférabilité et Accompagnement à l’adaptation des InteRventions en promotion de la santE (ASTAIRE) tool

Figure 3 indicates how the studies reported different aspects of the interventions, through the lens of the ASTAIRE tool. Results showed that, in the four categories upon which the tool is structured (population, environment, implementation, and support for transfer), the data reporting in the papers was very limited. The ASTAIRE items most often reported were “Communication of elements needed for transfer” (32% of articles), “Epidemiologic and socioeconomic characteristics” (31%), and “Institutional environment directly influencing intervention” (29%). The most frequently omitted ASTAIRE item was “Mechanisms for motivating providers”. On the other hand, according to the TIDieR checklist analysis, although no case provided detailed information, authors generally described the procedures and materials used in the intervention, as well as locations and providers. Conversely, articles rarely reported whether the intervention was modified during its course (describing in that case the changes implemented), or whether any strategies were used to improve compliance with the intervention or implementation fidelity.

### Impact evaluation

The impact of the interventions was measured in several ways, as shown in Additional file 4, providing a more detailed description of the outcomes measures used. Here we present the main results in four subsections related to such outcomes measures.

#### Use of measures for vector control

Two studies considered the use of vector control methods when measuring the impact of interventions. In studying an intervention to support program managers, Valadez et al. [63] used as an outcome the possession of insecticide treated bed-nets and indoor residual spraying (proportion of people protected). They found that intervention districts exhibited significant reductions in use of insecticide treated bed-nets and indoor residual spraying (the change in coverage proportion ranged from 35.9% (95% *CI*: +/− 26.2%) to 49.6% (95% *CI*: +/− 6.7%). Vanlerberghe et al. [43] measured the insecticide-treated materials coverage achieved with an intervention consisting of container inspections and treatment, insecticide-treated curtains, and educational interventions. They used this variable to analyze whether using insecticide-treated window curtains could reduce *Aedes aegypti* levels, depending on coverage attained.

#### Vector control

Some studies used intermediate outcomes to measure the vector control impact of the intervention, even though most of the studies considered final outcomes. Intermediate outcomes were the number of positive containers with larvae and/or pupae by house [22, 23, 30], overall average of positive containers with larvae and/or pupae (C+/C) [22], percentage of water-filled containers positive for larvae and/or pupae [25, 33, 48], and catch basins positive for larvae and pupae [62]. The results from Espinoza-Gómez et al. [22] stress that using an EI, preceded by intersectoral integration with the community, had a significantly stronger effect on reducing breeding sites for *Aedes aegypti* than did chemical spraying. Escudero-Támara and Villareal-Amaris [23] found a reduction of the number of *Aedes* breeding sites after an EI. Raju et al. [25] found a significant reduction in the percentage of primary positive containers for *Aedes albopictus*, from 33 to 5% for tires, and from 42 to 8% for drums, after communities were mobilized in the *Aedes aegypti* control program for source reduction in the peri-urban district. Caprara et al. [48] found a reduction in small discarded water containers in the intervention clusters (100% elimination in all visited houses) following an ecohealth approach. Basso et al. [33] found that the percentage change in number of containers registered after the intervention (EI with an entomological survey) was thoroughly different among six clusters studied, showing reductions between 26.1% (cluster 4) and 66.5% (cluster 20) in 17 clusters and increases in three clusters ranging from 9.4% (cluster 6) and 53.2% (cluster 13). The study by Pacheco-Coral et al. [30] revealed only partial success for the strategy in La Dorada (Colombia); houses that did not have larvae were characterized as having female heads of household or residents who were knowledgeable about larvae and the mode of transmission of dengue. Lastly, Ocampo et al. [62] found a higher percentage of catch basins positive for pupae during the pre-intervention period compared to the intervention period (71.4% versus 57.6%).

Concerning final outcomes, and in the case of dengue control, most studies used entomology indices, such as the pupae per person index (PPI) [33, 38, 43, 48–52, 57], the pupal index (PI) [33, 30], the pupae per hectare index (PHI) [33], the Breteau index [25, 33, 43, 45, 48, 57], the premises or house index (HI) [25, 33, 48, 57], the density of adult *Aedes aegypti* [34], the density of immature *Aedes aegypti* [34, 41], and the ovitrap index [24, 28]. The outcomes used for malaria control interventions were parasite rates [61] and the entomological inoculation rate [58]. Comparators between studies were diverse, and results were also heterogeneous with very variable magnitude. In all cases, the results were favourable to the analyzed intervention, with the exception of one study that reported the same effectiveness as the comparator [50].

#### Health measures

The incidence of illness was the health outcome used in four studies, including dengue-related illness [62], malaria [58], and cutaneous leishmaniasis [29, 42]. Ocampo et al. [62] found a reduction in dengue incidence (*RR* = 0.19) with an intervention based on community participation and mass control of vectors in street catch basins. Regarding malaria, Geissbühler et al. [58] concluded that larviciding reduced malaria infection risk among children ≤5 years of age (*OR* = 0.28) and provided protection at least as good as personal use of an insecticide-treated bed-net (*OR* = 0.76). Finally, in relation to leishmaniasis, Noazin et al. [42] evaluated an intervention based on installing deltamethrin-impregnated screens and curtains as a preventive measure for anthropologic cutaneous leishmaniasis (ACL). Their findings showed the short-term effectiveness of such preventive measures (*RR* = 0.84), but also highlighted the necessity of long-term sustainable strategies. Da Costa et al. [29] evaluated a strategy based on capture of sandflies and identification of vector species, chemical control through insecticide application, serological investigation and elimination of infected dogs, rodent control, and educational measures aimed at the local community. These results showed a 31.6% decrease in leishmaniasis cases (comparison pre-post intervention).

#### Social measures

Regarding social measures, studies measured interventions’ effects on people’s knowledge, attitudes, and practices, as well as population empowerment and participation in control activities conducted by intersectoral collaboration and at the household level. Outcomes used were knowledge of symptoms and transmission modalities [26, 27, 31, 55, 56], knowledge and beliefs about control measures [52], changes in people’s behaviour to control *Aedes aegypti* breeding sites [23–25], people’s abilities and adequate practices for controlling the vector [31], empowerment of prevention and control measures [26, 50, 52], household participation in dengue control activities [53], intersectoral participation in dengue control activities [53], and health worker capacity indicators [63]. All results from these studies were favourable to the evaluated intervention with a pre-post comparison with variable magnitudes. The impact outcomes in relation to study quality are presented in Fig. 3.

### Economic evaluation

#### Types of economic evaluation

Among the selected papers, three studies performed cost-effectiveness analyses [35, 40, 55], one a cost-benefit analysis [46], and one a cost-utility analysis [35]. All adopted a societal perspective, and Maheu-Giroux and Castro [55] and Baly et al. [40] also adopted a provider perspective (Tanzanian Ministry of Health and Social Welfare and Ministry of Health, respectively). The time horizons over which costs and consequences were evaluated ranged between 3 months [46] and 10 years [55], and the time horizons used by the other papers were 3 [35] and 5 years [40]. Only two papers used a discount rate for costs and outcomes, which was 3% [35, 55]. Three of the four articles included a sensitivity analysis to determine the robustness of the results [35, 46, 55].

#### Choice of health outcomes

The cost-effectiveness analyses used different measures. Maheu-Giroux and Castro [55] used three outcomes: malaria infections averted, malaria-associated deaths prevented, and disability-adjusted life years (DALY). Baly et al. [40] used the difference between the baseline averages of larval indices and *Aedes* foci numbers at baseline, and these average indices for the subsequent periods constituted the effectiveness measure. Shepard et al. [35] used quality adjusted life years (QALY) gained, taking into account the reduction in the number of yard and porch hours lost in a typical summer week due to mosquitoes. With regard to the cost-benefit and cost-utility analyses, monetary values were calculated from contingency valuations—in one of them directly by face-to-face interviews [35] and in the other by extracting values from the literature [46].

#### Estimated costs

For two papers, the costing sources were interviews [35, 46], a third used documents [55], and the last one used both interviews and documents [40]. With reference to the CHEERS checklist items, two studies had a high risk of bias [35, 40] and the other two had a low risk [46, 55]. Finally, although some elements, such as malaria incidence [55] or amount of rainfall [35], were identified as key factors that could affect an intervention, the results seemed to suggest that interventions performed by a combination of different stakeholders are more cost-effective than interventions performed by only one type of actor [40, 46].

### Sustainability analysis

Despite the lack of specific outcomes related to sustainability, the results presented here provide several avenues for improving intervention effectiveness and empowering sustainable effects. Specifically, 17 of the 42 papers addressed activities that, in line with the authors’ recommendations, could reinforce the sustainability of the vector control approaches. Based on these papers, we can consider three interrelated dimensions of sustainability in an IVM: social, economic, and environmental.

#### Social dimension related to sustainability

Although they were not the only studies to include community participation, in nine papers, all concerned with dengue, community involvement was the central axis of their preventive approach and/or vector control.

Community capacity-building was identified as the main component in the development of sustainability. In four papers, the increase in people’s knowledge about the prevention of vector transmission was articulated around community-based campaigns. In three of these studies the observational units were households. For instance, in a randomized community trial conducted by Espinoza-Gómez et al. [22], in which the educational campaign consisted of a series of house-by-house visits, community participation was found to be more effective than control methods such as insecticide spraying. Overall, the findings of this study provided evidence that an intervention that is personalized and based on the active participation of community leaders is more effective than the traditional vertical strategy of applying chemicals. The other two studies in this same line were pre–post evaluations of EIs. In the intervention by Pai et al. [24], the results showed the effectiveness of a short-term community-based cleanliness campaign, especially with regard to people’s knowledge and behaviour (surveyed) regarding vector transmission and prevention, which increased significantly from 57.4% before to 88.4%. after the campaign. The sources of dengue vectors were analyzed based on changes in the ovitrap index, which also decreased significantly, from 66.7% before the campaign to 39.3% 3 months afterward [24]. In the quasi-experimental study by Escudero-Támara and Villareal-Amaris [23], capacity-building was linked with a communication strategy—specifically, the main concepts of the Communication for Behavioural Impact (COMBI) toolkit. At the end of the intervention with each of the participant groups, 64.8% were classified as being in the action stage, and a significant reduction in intradomiciliary *Aedes* breeding sites, from 92.6 to 35.2% (*P* = 0.001), had been achieved [23]. Another study, also following a quasi-experimental design, focused on schoolchildren by using a game as a teaching–learning tool to impart knowledge and prevention skills about dengue; results showed that the children’s knowledge and skills, measured prior to the program (6.5 and 18.4 points, respectively) were higher in the final test (8.25 and 22.9 points, respectively; *P* < 0.05) [31].

The community-based trial conducted by Cáceres-Manrique et al. [26] showed the efficacy of social mobilization to improve knowledge and practices and highlighted its role in public empowerment with regard to preventive and control measures—key elements to ensure the sustainability of community-based strategies. Similarly, the study by Toledo et al. [44] evaluated the sustainability of an intervention strategy to achieve active participatory control over a two-year period. Their results suggested that enhancing the sustainability of community-based strategies requires, at the very least, institutionalizing the provision of basic sanitation services, but also engaging in collaborations with other community partners and creating coordinating groups to strengthen community involvement.

In connection with the above points, our review also highlighted the results of implementing an eco-bio-social approach focused on the community. Three studies were designed following this approach [49–51]. Their results provided evidence of the positive influence of establishing a collaboration framework among local governments, program planners, and communities. In the multi-country study by Sommerfeld et al. [49], the results showed that vector management was more sustainable when it complemented or replaced other interventions by: a) targeting water container interventions that achieved a significant reduction in vectors; b) using novel non-insecticidal intervention tools; and c) involving different partners. Following the same approach, Wai et al. [40] emphasized that, for sustainability and population empowerment, a key element is the connection established between the different interest groups. The project conducted by Mitchell-Foster et al. [41], which compared the effectiveness and feasibility of scaling up an eco-bio-social approach for implementing an integrated community-based dengue prevention strategy to the effectiveness of existing insecticide-based approaches, also suggested that ecosystems approaches to vector-borne diseases such as dengue are primarily successful when elements of social mobilization are incorporated, together with intersectoral collaborations.

#### Economic dimension related to sustainability

Six papers addressed various components that contributed to understanding sustainability-building from an economic perspective. In two of these, resource optimization was the key element, manifested in one by the integration of two programs [27] and in the other through a combination of interventions, such as indoor residual spraying and the use of long-lasting insecticidal bed-nets [64].

Three other studies were specifically designed to determine the cost of public sector vector control programs, but they also provided findings on sustainability from the cost analysis standpoint [36, 45, 47]. Thus, Packierisamy et al. [36] studied differences in costs and resource consumption between district health departments (DHDs) and local authorities (LAs) and found that inspection services, fogging, and larviciding aimed at dengue vector control by LAs were more cost-efficient than the approaches developed by the DHDs. Thalagala et al. [45] confirmed that public sector costs of dengue control activities and direct costs of hospitalizations imposed a significant economic burden in contexts such as Sri Lanka; specifically, the total public sector cost of dengue control activities in the district studied during the epidemic year of 2012 was estimated at US$971 360, indicating a per capita cost of US$0.42 for dengue control activities. These findings underscore the importance of prevention activities to reduce patient numbers and strengthen health system sustainability. Baly et al. [47] found that the annual per-household cost documented by their study was higher than the per-household cost of most routine *Aedes* control programs worldwide. Thus, program sustainability was connected with conventional routinization activities, mainly related with insecticide use.

Finally, the intervention program by Samuelsen et al. [54], the only one that used both entomological and anthropological approaches to examine sociocultural aspects of a mosquito control intervention, showed that the most commonly used method (the coil) for the prevention of mosquito nuisance in Burkina Faso was the one the local population considered not only least expensive but also least effective. Their findings prompted them to suggest that processes aimed at ensuring economic sustainability must to take into consideration that the choice of control measures by the local population and by the system as a whole is primarily based on financial accessibility (Table 2).

**Table 2:** Descriptions of economic evaluation studies

Study	Intervention	Study design	Comparison	Variable of interest	Time horizon
Maheu-Giroux & Castro [45]	Larviciding, vector surveillance and control	CE	Three different scenarios (incidences)	Infections averted, deaths prevented and DALY	10 years
Orellano & Pedroni [36]	Fogging plus control of immature mosquitoes	CB	Control: areas without intervention	CV	3 months
Baly et al. [28]	Entomological surveillance, inspection, larviciding, adulticiding, health education, and control legislation	CE	Control: areas without intervention	Larval indices	3 years
Shepard et al. [24]	Education campaigns, larviciding and adulticiding	CB & CU	Control: areas without intervention	CB: CV CU: QALY	5 years

*CE* Cost-effectiveness, *CB* Cost-benefit, *CU* Cost-utility, *QALY* Quality adjusted life years, *CV* Monetary terms, calculated by contingency valuations, *DALY* Disability-adjusted life years

#### Environmental dimension related to sustainability

Four papers provided elements for understanding sustainability from an environmental perspective, although with clearly related implications for social dimensions. Wai et al. [50] conducted an intervention study to build a partnership-driven ecosystem management intervention and then to analyze its feasibility, process, and effectiveness in reducing vector densities. Their results showed that, for sustainability, the partnership approach with targeted container interventions was found to be superior to the vertical approach. Along the same lines, Samuelsen et al. [54] found not only that reducing VBD-related morbidity and mortality requires a combination of biolarvicide treatment and insecticide treated nets, but also that, to enhance sustainability, such combinations must take into account local sociocultural factors.

Only one study intervention was based on regular application of microbial larvicides using a community-based but vertically managed approach. This procedure, developed to reduce malaria prevalence and as a complement to the existing protection provided by insecticide-treated bed-nets, was considered a useful IVM strategy, especially in rapidly growing urban settings [58]. Another article called attention to the design of covers or biological control methods for water storage containers as a relevant component of the sustainability of a control program [25].

## Discussion

WHO recommends IVM as the main strategy for vector control and encourages member states to adopt this approach [5]. However, many VBD-endemic countries have not adopted this strategic framework to guide their actions [12, 65]. This scoping review was intended to provide information to support the development of strategies based on this approach. We have also highlighted the implications and the identified gaps in terms of both future research and public health policy and/or practice around this issue.

This study shows that evidence on IVM integration in endemic countries, and particularly in Africa, is limited. From a methodological standpoint, half of the studies in this review had performed a quantitative descriptive analysis, reflecting the need to produce evidence based on more robust quantitative methods; in this respect, randomized controlled trials could be an option. We also identified a significant gap in the use of qualitative methods within this field of study, especially to understand the process of intervention implementation. Finally, there is a major lack of evidence regarding the economic evaluation of IVM.

The MMAT analysis [18] revealed variability in the quality of available evidence. In this regard, it should be noted that our descriptions of quality are based on how the authors presented their study; in certain cases, the information available in the paper was insufficient to answer some of the questions. Similarly, many papers did not provide the information needed to complete the TIDieR checklist and the ASTAIRE tool. This raises the need for authors to provide more detailed information to characterize their interventions, to help in assessing their transferability and whether results can be extrapolated from one setting to another. In this respect, scientific journals have a key role to play: to advance the field of population health intervention research (PHIR), they will need to publish more intervention research and related case studies [66].

The main limitation of our review is related to the studies’ approaches, given that the term “integrated vector management” itself refers to a framework or a strategy, rather than to a particular procedure. In many cases, this made it difficult to decide whether a study had been designed within this approach. This may have limited the number of papers considered for the final phase of analysis. Like other scoping reviews, our study is subject to the same limitations as any literature review, which include the potential omission of relevant sources of information and the review being dependent on the information available (publication bias). Moreover, because our subject is very much linked to public policies, there is potential for positive-results bias, meaning that authors (and journals) are more likely to publish positive results than negative or inconclusive findings. On the other hand, another limitation of this scoping review is that new tools for vector control are not addressed in detail. It is important to point out that several new tools are undergoing entomological trials, but only a few are brought under epidemiological evaluations of efficacy. In this sense, it is necessary to highlight the key role that the Vector Control Advisory Group of WHO is playing in facilitating rigorous evaluation and testing of new tools and technologies.

### Implications for future research

Our results highlighted the difficulty of comparing the different studies in the review given the diversity of interventions and components addressed, as well as the heterogeneity within each study. Our findings showed that, for successful community involvement, it is critical to take into account the intervention’s contextual elements. The literature revealed the importance, for decision-makers, of performing pilot studies before implementation, so that programs can be adapted to the context and relevant human factors [67]. However, such contextualization of procedures should not impede the standardized application of certain instruments and research methods related to the different sciences working on vector control and prevention [49].

In recent years, the social determinants of health framework has furthered the debate on the relationship between inequalities and disease, in particular favouring approaches that take into account the conditions in which people’s lives unfold. This framework has not only led indirectly to the examination of social relations as health determinants, but has also generated a corpus of knowledge that provides greater understanding of inequalities within global health processes [68]. The results of this review showed, however, that this theoretical framework is rarely taken into consideration in the formulation of studies about vector control. Especially evident was the absence of any consideration of health inequalities in the interpretation of results. Yet this approach may provide a better understanding of non-biological factors, such as determinants that depend on cultural and socioeconomic conditions, related to vector-borne morbidity and mortality [69]. Therefore, we consider that to advance further in this field the researchers should take into account to a greater extent how action in different sectors is affected by the particular political, social and cultural contexts, and how the complex mix of politics, economic development, culture and collective action interact to influence the population health outcomes. This requires extend the existing knowledge by exploring the influence of the broader determinants of health using innovative mixed methods.

The results of this review emphasize the need to generate more scientific evidence, leveraging all the potential offered by the different evaluation designs in public health [70]. In this regard, what emerges from our analyses as a notable deficiency is the dearth of health policy evaluations focusing on effectiveness assessment using data from real-world conditions [71]. Research methods also need to be strengthened to support evidence-based decision-making that takes into account local conditions of diseases and disease transmission. Mixed methods combining quantitative, qualitative, and participatory techniques can be a good way of taking into account all sectors involved and analyzing the side-effects of strategies [72].

Particularly relevant for the IVM approach are aspects related to economic assessments and sustainability. Although the problem of VBDs has been worsening globally in recent years, we found few economic evaluations that met our inclusion criteria. This is another key line of research that should be strengthened; given the scarcity of resources, economic evaluation is becoming more important as a tool to inform resource allocation by comparing various alternatives for action in terms of their costs and effects on health [73]. Many studies used intermediate outcomes as a measure of effectiveness, but this can lead to suboptimal recommendations. Ideally evaluations should focus on final health outcomes [16, 19]. Along the same lines, to monitor the sustainability of interventions, longitudinal research is required over an extended period. In particular, evidence is needed on the environmental sustainability of interventions.

Most studies that met the inclusion criteria for the review were focused on dengue. This highlights the need to implement integrated control strategies for other diseases. In this regard, researchers might consider adopting a multi-disease approach to IVM, that is, taking into account all prevalent VBDs within one control strategy [4].

**Table:** 

Knowledge gaps and priority needs for future research	
• Researchers need to provide more detailed characterizations of interventions and their processes, so that their transferability can be better assessed. • Researchers need to produce more evidence based on randomized trials (cluster) and the use of qualitative and mixed methods. • Researchers need to identify more health outcomes when evaluating programs. • More research is needed on integrated approaches with assessments of impacts on entomological and health outcomes. • Economic evaluation studies with a long time horizon are needed. • More research is needed that takes into consideration the social determinants of health. • Research is needed to increase understanding of VBDs from the standpoint of health inequalities. • More studies are needed that provide in-depth descriptions of the pathways or mechanisms through which context and intervention influence population health. • More studies are needed that monitor the sustainability of interventions over the long term. • More studies are needed that consider the incidence of illness as a key factor to determine the cost-effectiveness of an intervention in a specific context. • Research is needed to calculate affordability, once cost-effectiveness is ensured, through budget impact analysis.	

### Implications for public health policy and practice

IVM has impacts on reducing breeding sites [22, 23, 25, 30, 33, 48, 62], improving the entomology index [24, 25, 28, 30, 33, 34, 38, 41, 43, 45, 48–52, 57], and lowering parasite rates [61]. The results in the papers we reviewed were heterogeneous with variable magnitudes, although in all cases they were favourable to the intervention. Evidence related to IVM’s impact on health outcomes was very limited [29, 42, 58, 62].

Achieving sustainability is one of the major current challenges in VBD control programs [17]. Our findings showed that, to foster sustainability, interventions must focus especially on capacity building in the recipient community. According to the studies we reviewed, this element is generally understood as the interaction between human capital, organizational resources, and social capital within a given context that can be leveraged to solve collective problems and improve or maintain the community’s well-being [74, 75]. Thus, in top-down programs the capacity for sustainability requires both organizational capability and people’s expertise. Attempting to implement a community participation process to gather support for program activities without capacity building and real active community involvement may be one of the clearest ways to create an unsustainable initiative. From a public health perspective, among other implications for practice, this should lead to a growing interest in the application of participatory research methods to generate greater mobilisation and community interest on health determinants, increasing the empowerment and social change potential of the interventions [76].

An intervention’s effectiveness will depend on many factors. Especially important are social mobilization to achieve long-lasting behaviours, the durability of materials used for the interventions, and the coverage attained or the specific environmental conditions. Likewise, an intervention’s effectiveness also depends on people’s positive perceptions of the control methods used, always keeping in mind, however, that their choice of method could be based primarily on financial accessibility rather than perceived effectiveness [54]. IVM interventions need to take into account local sociocultural factors. While it remains a challenge to involve local urban populations in control efforts and prevention activities, our findings stress that any measures adopted should be based more on community involvement than on vertical approaches [22].

Within this field of intervention research, planning for sustainability is a core issue in implementing processes for improving population health [74, 76]. The literature suggests that, to promote sustainability, it is essential to focus on the routinization of activities resulting from a program. Therefore, maintaining the health benefits achieved involves more than just continuing an intervention or program; a host organization is also needed to continue the program’s activities [77]. As such, institutionalization is also a key process on the path toward sustainability. Along these lines, one relevant factor influencing the impact and sustainability of a community-based approach to vector control could be the municipal provision of sanitation services [44]. Given the importance of the institutionalization process, another key IVM-related strategy to foster the sustainability of community-based strategies is to promote intersectoral coordination [49]. Our results provided evidence of the positive impacts of collaboration among communities, local governments, and program planners. This underscores the importance, especially in urban endemic zones, of integrating the efforts and resources of all actors involved with prevention and control strategies. In the case of research-based interventions, this reinforces the need for seeing sustainability as a further stage of the implementation process [17, 78].

From an environmental standpoint, the results also underlined the need to consider the different elements that can influence a program’s sustainability in a specific urban area. For instance, the results from Shepard et al.’s [35] study of an integrated pest management program to control the Asian tiger mosquito showed that climatic conditions can negatively influence an intervention when planners do not anticipate the potential impact of the rainy season in a given urban context. Similarly, impact assessment of entomological and clinical parameters is also relevant for the future of integrated approaches. This may help identify relationships between larval control, environmental management, and chemicals used [32]. In any case, as interventions become more effective, we can assume there will be less need for chemical products. This is considered a relevant environmental outcome because it could translate into lower vector resistance to those products. Among other considerations, this reinforces the ‘One Health’ approach developed by the WHO to designing and implementing programmes, policies, legislation and research in which different sectors, such as public health, animal health, plant health and the environment, work together to achieve better population health outcomes [79].

This research determined the importance of designing, developing, and analyzing multi-partnership interventions with an emphasis on community participation. Related to this approach, within an IVM strategy, eco-bio-social research could be considered an important framework for systematic assessment of vector control needs and for developing partnership strategies at the local level [49].

**Table:** 

Implications for public health policy and/or practice	
• Interventions should be mainly based on community involvement. • Interventions must be specially focused on community capacity building. • Interventions must be tailored to local sociocultural factors. • Less use of chemical products is considered a relevant environmental outcome. • Interventions must take into account the specific environmental conditions and aspects such as social mobilization activities to achieve more lasting behaviours, increase the durability of materials used, or enhance the coverage attained. • Institutionalization must also be a key process leading to sustainability, combined with conventional routinization activities such as provision of basic sanitation services. • Programs undertaken by local authorities may be more efficient than those developed at a more aggregated level. • Planners could adopt a multi-disease approach to IVM. • The community involvement approach requires sociocultural contextualization of interventions. • Researchers could adopt a multi-disease approach to IVM.	

## Conclusions

IVM has an impact on reducing vector breeding sites and improving the entomology index, whereas evidence of health outcomes impact is limited. Social outcomes of IVM are improvement of abilities and capacities, empowerment, and community knowledge. Economic evaluations are scarce, and cost-effectiveness is dependent on disease incidence. Community capacity building is the main component of sustainability, together with a collaboration framework, institutionalization, and routinization of activities.

## Additional files

Additional file 1: Translation of the abstract into the five official working languages of the United Nations. (PDF 705 kb)

Additional file 2: Complete search strategy by database. (DOCX 31 kb)

Additional file 3: Description of selected studies. (DOCX 66 kb)

Additional file 4: Description of outcome measures for impact. (DOCX 77 kb)

## Abbreviations

ASTAIRE: AnalySe de la Transférabilité et Accompagnement à l’adaptation des InteRventions en promotion de la santE
CHEERS: Consolidated Health Economic Evaluation Reporting Standards
DALY: Disability-adjusted life years
IVM: Integrated vector management
MeSH: Medical Subject Headings
MMAT: Mixed Method Appraisal Tool
TIDieR: Template for Intervention Description and Replication
UMCP: Urban Malaria Control Program
VBD: Vector-borne diseases
WHO: World Health Organization

## References

[1] WHO (2014). A global brief on vector-borne diseases.

[2] Beier JC, Keating J, Githure JI, Macdonald MB, Impoinvil DE, Novak RJ (2008). Integrated vector management for malaria control. Malar J.

[3] Chanda E, Masaninga F, Coleman M, Sikaala C, Katebe C, Macdonald M (2008). Integrated vector management: the Zambian experience. Malar J.

[4] Grepin KA, Reich MR (2008). Conceptualizing integration: a framework for analysis applied to neglected tropical disease control partnerships. PLoS Negl Trop Dis.

[5] WHO (2004). Global strategic framework for integrated vector management.

[6] Leach-Kemon K, Chou DP, Schneider MT, Tardif A, Dieleman JL, Brooks BPC (2012). The global financial crisis has led to a slowdown in growth of funding to improve health in many developing countries. Health Aff (Millwood).

[7] Glonti K, Gordeev VS, Goryakin Y, Reeves A, Stuckler D, McKee M (2015). A systematic review on health resilience to economic crises. PLoS One.

[8] WHO (2012). Making a difference: TDR strategic plan 2012-2017.

[9] Golding N, Wilson AL, Moyes CL, Cano J, Pigott DM, Velayudhan R (2015). Integrating vector control across diseases. BMC Med.

[10] WHO (2012). Handbook for integrated vector management.

[11] United Nations (2014). World urbanization prospects.

[12] Okia M, Okui P, Lugemwa M, Govere JM, Katamba V, Rwakimari JB (2016). Consolidating tactical planning and implementation frameworks for integrated vector management in Uganda. Malar J.

[13] Schwartz F, Bitzer E, Long A, Bitzer E (1997). A systems perspective of evaluation in health care. Health outcomes and evaluation: context, concepts and successful applications. European Clearing Houses on Health Outcomes, Nuffield Institute for Health.

[14] Banta D, Long A, Bitzer E (1997). Health outcomes and evaluation: context, concepts and successful applications. Health outcomes and evaluation: context, concepts and successful applications. European Clearing Houses on Health Outcomes. Nuffield I. Nuffield: University of Leeds.

[15] Konu A, Rissanen P, Ihantola M, Sund R (2009). “Effectiveness” in Finnish healthcare studies. Scand J Public Health.

[16] Drummond M, O’Brien B, Stoddart G, Torrance G (1997). Methods for the economic evaluation of health care programmes.

[17] Scheirer MA, Dearing JW (2011). An agenda for research on the sustainability of public health programs. Am J Public Health.

[18] Pace R, Pluye P, Bartlett G, Macaulay AC, Salsberg J, Jagosh J (2012). Testing the reliability and efficiency of the pilot Mixed Methods Appraisal Tool (MMAT) for systematic mixed studies review. Int J Nurs Stud.

[19] Husereau D, Drummond M, Petrou S, Carswell C, Moher D, Greenberg D (2013). Consolidated Health Economic Evaluation Reporting Standards (CHEERS) statement. Value Health.

[20] Hoffmann TC, Glasziou PP, Boutron I, Milne R, Perera R, Moher D (2014). Better reporting of interventions: template for intervention description and replication (TIDieR) checklist and guide. BMJ.

[21] Cambon L, Minary L, Ridde V, Alla F (2014). A tool to facilitate transferability of health promotion interventions: ASTAIRE. Sante Publique.

[22] Espinoza-Gomez F, Hernandez-Suarez CM, Coll-Cardenas R (2002). Educational campaign versus malathion spraying for the control of *Aedes aegypti* in Colima, Mexico. J Epidemiol Community Health.

[23] Escudero-Tamara E, Villareal-Amaris G (2015). Educational intervention for the control of dengue in family environments in a community in Colombia. Rev Peru Med Exp Salud Publica.

[24] Pai H-H, Hong Y-J, Hsu E-L (2006). Impact of a short-term community-based cleanliness campaign on the sources of dengue vectors: an entomological and human behavior study. J Environ Health.

[25] Raju A (2003). Community Mobilization in Aedes aegypti Control Programme by source reduction in Peri-Urban District of Lautoka, Viti Levu, Fiji Islands. Dengue Bull.

[26] Caceres-Manrique F de M, Angulo-Silva ML, Vesga-Gomez C (2010). Efficacy of the social mobilization and the social participation in dengue control measures. Biomedica.

[27] Chiaravalloti Neto F, Barbosa AAC, Cesarino MB, Favaro EA, Mondini A, Ferraz AA (2006). Dengue control in an urban area of Brazil: impact of the family health program on traditional control. Cad Saude Publica.

[28] Fonseca DM, Unlu I, Crepeau T, Farajollahi A, Healy SP, Bartlett-Healy K (2013). Area-wide management of Aedes albopictus. Part 2: gauging the efficacy of traditional integrated pest control measures against urban container mosquitoes. Pest Manag Sci.

[29] Da Costa CM, Moutinho FFB, Bruno SF (2004). A experiência do município de Paraty (Rio de Janeiro, Brasil) na prevenção e controle da leishmaniose tegumentar americana. Parasitol Latinoam.

[30] Pacheco-Coral Adel P, Quinones-Pinzon ML, Serrato-Pomar IM, Rivas-Munoz FA (2010). Evaluating an information, education and communication (IEC) strategy which was adopted for *Aedes aegypti* control in La Dorada, Colombia. Rev Salud Publica (Bogota).

[31] Vivas E, Guevara De Sequeda M (2003). A game as an educational strategy for the control of Aedes aegypti in Venezuelan schoolchildren. Rev Panam Salud Publica.

[32] Skovmand O, Ouedraogo TDA, Sanogo E, Samuelsen H, Toe LP, Bosselmann R (2011). Cost of integrated vector control with improved sanitation and road infrastructure coupled with the use of slow-release Bacillus sphaericus granules in a tropical urban setting. J Med Entomol.

[33] Basso C, Garcia da Rosa E, Romero S, Gonzalez C, Lairihoy R, Roche I (2015). Improved dengue fever prevention through innovative intervention methods in the city of Salto, Uruguay. Trans R Soc Trop Med Hyg.

[34] Ocampo CB, Gonzalez C, Morales CA, Perez M, Wesson D, Apperson CS (2009). Evaluation of community-based strategies for Aedes aegypti control inside houses. Biomedica.

[35] Shepard DS, Halasa YA, Fonseca DM, Farajollahi A, Healy SP, Gaugler R (2014). Economic evaluation of an area-wide integrated pest management program to control the Asian tiger mosquito in New Jersey. PLoS One.

[36] Packierisamy PR, Ng C-W, Dahlui M, Venugopalan B, Halasa YA, Shepard DS (2015). The cost of dengue vector control activities in Malaysia by different service providers. Asia Pacific J Public Health.

[37] Gurtler RE, Garelli FM, Coto HD (2009). Effects of a five-year citywide intervention program to control *Aedes aegypti* and prevent dengue outbreaks in northern Argentina. PLoS Negl Trop Dis.

[38] Quintero J, Garcia-Betancourt T, Cortes S, Garcia D, Alcala L, Gonzalez-Uribe C (2015). Effectiveness and feasibility of long-lasting insecticide-treated curtains and water container covers for dengue vector control in Colombia: a cluster randomised trial. Trans R Soc Trop Med Hyg.

[39] Betancourt Betancourt JA, García Rodríguez JF, Alfonso PJ, Llambias Peláez JJ, García Fariñas A (2011). Análisis de eficiencia relativa en el control del Aedes aegypti del municipio Camagüey. Rev Arch Médico Camagüey.

[40] Baly A, Toledo ME, Vanlerberghe V, Ceballos E, Reyes A, Sanchez I (2009). Cost-effectiveness of a community-based approach intertwined with a vertical Aedes control program. Am J Trop Med Hyg.

[41] Ordonez Gonzalez JG, Thirion J, Garcia Orozco A, Rodriguez AD (2011). Effectiveness of indoor ultra-low volume application of Aqua Reslin(R) Super during an emergency. J Am Mosq Control Assoc.

[42] Noazin S, Shirzadi MR, Kermanizadeh A, Yaghoobi-Ershadi M-R, Sharifi I (2013). Effect of large-scale installation of deltamethrin-impregnated screens and curtains in Bam, a major focus of anthroponotic cutaneous leishmaniasis in Iran. Trans R Soc Trop Med Hyg.

[43] Vanlerberghe V, Villegas E, Oviedo M, Baly A, Lenhart A, McCall PJ (2011). Evaluation of the effectiveness of insecticide treated materials for household level dengue vector control. PLoS Negl Trop Dis.

[44] Toledo Romani ME, Vanlerberghe V, Perez D, Lefevre P, Ceballos E, Bandera D (2007). Achieving sustainability of community-based dengue control in Santiago de Cuba. Soc Sci Med.

[45] Thalagala N, Tissera H, Palihawadana P, Amarasinghe A, Ambagahawita A, Wilder-Smith A (2016). Costs of Dengue control activities and hospitalizations in the public health sector during an epidemic year in urban Sri Lanka. PLoS Negl Trop Dis.

[46] Orellano PW, Pedroni E (2008). Cost-benefit analysis of vector control in areas of potential dengue transmission. Rev Panam Salud Publica.

[47] Baly A, Gonzalez K, Cabrera P, Popa JC, Toledo ME, Hernandez C (2016). Incremental cost of implementing residual insecticide treatment with delthametrine on top of intensive routine Aedes aegypti control. Tropical Med Int Health.

[48] Caprara A, Lima JWDO, Peixoto ACR, Motta CMV, Nobre JMS, Sommerfeld J (2015). Entomological impact and social participation in dengue control: a cluster randomized trial in Fortaleza, Brazil. Trans R Soc Trop Med Hyg.

[49] Sommerfeld J, Kroeger A (2012). Eco-bio-social research on dengue in Asia: a multicountry study on ecosystem and community-based approaches for the control of dengue vectors in urban and peri-urban Asia. Pathog Glob Health.

[50] Wai KT, Htun PT, Oo T, Myint H, Lin Z, Kroeger A (2012). Community-centred eco-bio-social approach to control dengue vectors: an intervention study from Myanmar. Pathog Glob Health.

[51] Mitchell-Foster K, Ayala EB, Breilh J, Spiegel J, Wilches AA, Leon TO (2015). Integrating participatory community mobilization processes to improve dengue prevention: an eco-bio-social scaling up of local success in Machala, Ecuador. Trans R Soc Trop Med Hyg.

[52] Tana S, Umniyati S, Petzold M, Kroeger A, Sommerfeld J (2012). Building and analyzing an innovative community-centered dengue-ecosystem management intervention in Yogyakarta, Indonesia. Pathog Glob Health.

[53] Kittayapong P, Thongyuan S, Olanratmanee P, Aumchareoun W, Koyadun S, Kittayapong R (2012). Application of eco-friendly tools and eco-bio-social strategies to control dengue vectors in urban and peri-urban settings in Thailand. Pathog Glob Health.

[54] Samuelsen H, Toe LP, Baldet T, Skovmand O (2004). Prevention of mosquito nuisance among urban populations in Burkina Faso. Soc Sci Med.

[55] Maheu-Giroux M, Castro MC (2014). Cost-effectiveness of larviciding for urban malaria control in Tanzania. Malar J.

[56] Maheu-Giroux M, Castro MC (2013). Do malaria vector control measures impact disease-related behaviour and knowledge? Evidence from a large-scale larviciding intervention in Tanzania. Malar J.

[57] Vanlerberghe V, Toledo ME, Rodriguez M, Gomez D, Baly A, Benitez JR (2009). Community involvement in dengue vector control: cluster randomised trial. BMJ.

[58] Geissbuhler Y, Kannady K, Chaki PP, Emidi B, Govella NJ, Mayagaya V (2009). Microbial larvicide application by a large-scale, community-based program reduces malaria infection prevalence in urban Dar es Salaam, Tanzania. PLoS One.

[59] Ogoma SB, Kannady K, Sikulu M, Chaki PP, Govella NJ, Mukabana WR (2009). Window screening, ceilings and closed eaves as sustainable ways to control malaria in Dar es Salaam, Tanzania. Malar J.

[60] Chaki PP, Mlacha Y, Msellemu D, Muhili A, Malishee AD, Mtema ZJ (2012). An affordable, quality-assured community-based system for high-resolution entomological surveillance of vector mosquitoes that reflects human malaria infection risk patterns. Malar J.

[61] Caldas de Castro M, Yamagata Y, Mtasiwa D, Tanner M, Utzinger J, Keiser J (2004). Integrated urban malaria control: a case study in dar es salaam, Tanzania. Am J Trop Med Hyg.

[62] Ocampo CB, Mina NJ, Carabali M, Alexander N, Osorio L (2014). Reduction in dengue cases observed during mass control of *Aedes* (Stegomyia) in street catch basins in an endemic urban area in Colombia. Acta Trop.

[63] Valadez JJ, Devkota B, Pradhan MM, Meherda P, Sonal GS, Dhariwal A (2014). Improving malaria treatment and prevention in India by aiding district managers to manage their programmes with local information: a trial assessing the impact of lot quality assurance sampling on programme outcomes. Tropical Med Int Health.

[64] Das M, Banjara M, Chowdhury R, Kumar V, Rijal S, Joshi A (2008). Visceral leishmaniasis on the Indian sub-continent: a multi-centre study of the costs of three interventions for the control of the sandfly vector, *Phlebotomus argentipes*. Ann Trop Med Parasitol.

[65] Mnzova A, Williams J, Bos R, Zaim M (2011). Implementation of integrated vector management for disease vector control in the Eastern Mediterranean: where are we and where are we going?. East Mediterr Health J.

[66] Di Ruggiero E, Potvin L, Allegrante JP, Dawson A, De Leeuw E, Dunn JR (2017). Ottawa statement from the sparking solutions summit on population health intervention research. Can J Public Health.

[67] Belaid L, Ridde V (2015). Contextual factors as a key to understanding the heterogeneity of effects of a maternal health policy in Burkina Faso?. Health Policy Plan.

[68] Marmot M, Allen J, Goldblatt P (2010). A social movement, based on evidence, to reduce inequalities in health. Soc Sci Med.

[69] Carabali M, Hernandez LM, Arauz MJ, Villar LA, Ridde V (2015). Why are people with dengue dying? A scoping review of determinants for dengue mortality. BMC Infect Dis.

[70] Victora CG, Habicht J-P, Bryce J (2004). Evidence-based public health: moving beyond randomized trials. Am J Public Health.

[71] Campbell M, Fitzpatrick R, Haines A, Kinmonth AL, Sandercock P, Spiegelhalter D (2000). Framework for design and evaluation of complex interventions to improve health. BMJ.

[72] Kemm J (2001). Health impact assessment: a tool for healthy public policy. Health Promot Int.

[73] Davies L, Drummond M, Papanikolaou P (2000). Prioritizing investments in health technology assessment. Can we assess potential value for money?. Int J Technol Assess Health Care.

[74] Gruen RL, Elliott JH, Nolan ML, Lawton PD, Parkhill A, McLaren CJ (2008). Sustainability science: an integrated approach for health-programme planning. Lancet (London, England).

[75] Chaskin RJ (2001). Building community capacity: a definitional framework and case studies from a comprehensive community initiative. Urban Aff Rev.

[76] Baum F (2016). Power and glory: applying participatory action research in public health. Gac Sanit.

[77] Proctor E, Luke D, Calhoun A, McMillen C, Brownson R, McCrary S (2015). Sustainability of evidence-based healthcare: research agenda, methodological advances, and infrastructure support. Implement Sci.

[78] Pluye P, Potvin L, Denis JL, Pelletier J (2004). Program sustainability: focus on organizational routines. Health Promot Int.

[79] Wiltsey Stirman S, Kimberly J, Cook N, Calloway A, Castro F, Charns M (2012). The sustainability of new programs and innovations: a review of the empirical literature and recommendations for future research. Implement Sci.

