# Supplementary material for: Impact, economic evaluation, and sustainability of integrated vector management in urban settings to prevent vector-borne diseases: a scoping review
Source: Infect Dis Poverty. 2018 Sep 3;7:83. doi: 10.1186/s40249-018-0464-x (PMC6120095; doi:10.1186/s40249-018-0464-x)
Supplement: Supplementary file 1 — Translation of the abstract into the five official working languages of the United Nations. (PDF 705 kb) [file 40249_2018_464_MOESM1_ESM.pdf]

Translation of the abstract into the five official working languages of the United Nations

التأثير والتقويم الاقتصادي والاستدامة للإدارة المتكاملة لناقلات الأمراض في المناطق الحضرية لمنع الأمراض المنقولة: مراجعة دراسية

جورج ماركوس-ماركوس‘ أنطونيو أورلي دي لابري-ليما، سيلفيا تورو كارديناس، مارينا لاكاسانا، ستيفاني ديغروت، فاليري رايد، كلارا بيرموديز-تامايو

الملخص

لمحة: مراقبة أمراض التي تنقلها النواقل (VBD) هي إحدى التحديات الكبرى لدى أعمال الصحة العالمية. أدى التوسع الحضري السريع وغير المنضبط إلى زيادة الإهتمام في معالجة هذه التحديات بواسطة اسلوب الإدارة الكاملة لناقلات الأمراض (IVM). وكان الهدف تعريف العناصر المتعلقة بالتأثيرات والتقويم الإقتصادي والاستدامة التي قد تساهم في هذا الأسلوب المتكامل لمنع أمراض التي تنقلها النواقل.

الجزء الرئيس: أجرينا مراجعةً دراسيةً للمطبوعات المتاحة من 2000 إلى 2016 بالاستعانة بمواقع مثل: محرك البحث ببمد PubMed ومواقع العلوم وكوركرين ochraneC وسنهال CINHAL وإيكنليتEconlit وليليكاس LILACS وقاعدة بيانات الصحة العالمية وسكوبس Scopus وإمبيس Embase وكذلك نشرة الأمراض المداريّة وقاعدة بيانات مكتبة منظمة الصحة العالمية و برنامج منظمة الصحة العالمية لتقييم مبيدات الآفات والباحث العلمي لقوقل. وتم إستخدام مصطلحات من مختصر العناوين الرئيسية للمواضيع الطبية ومصطلحات حرة. وتم إستعمال نموذج لاستخراج البيانات يحتوي على TIDier و ASTAIRE. وكذلك MMAT و CHEERS لتقويم الجودة. ومن بين 42 ملفاً تمت مراجعتها، ركز 30 على حمى الدنك، و 8 على الملاريا، و 2 على مرض الليشمانيات. وأكثر من نصف الدراسات تم إجراؤها في الأمريكيتين. استخدم نصفها منهجاً وصفياً كمياً (n = 21)، تم إتباعه بتجارب عشوائية عنقودية مراقَبة (n = 11). بخصوص التأثيرات كانت النتائج: أ- استعمال إجراءات لمراقبة حامل المرض، ب- مراقبة حامل المرض، ج- إجراءات صحية، د- إجراءات إجتماعية. حدت الإدارة الكاملة لناقلات الأمراض من أماكن التكاثر ومؤشر الحشرات ومعدل الطُفيليات. كانت النتائج متنوعةً وذات مقادير مختلفة، لكنها في كل الحالات كانت مناسبةً للتدخل. وكانت النتائج الاجتماعية تحسين القدرات والطاقات والتمكين والمعرفة المجتمعية. أما بشأن التقويم الاقتصادي، فقد قامت أربع دراسات فقط بإجراء تحليل اقتصادي، وتغلبت منافع التدخل على التكاليف. وكانت الفعالية من حيث التكلفة معتمدةً على انتشار المرض. وقدمت النتائج عناصر أساسيةً لتحليل الاستدامة من ناحية ثلاثة أبعاد (الاجتماعي، والاقتصادي، والبيئي) مؤكدةً على تطبيق منهج اجتماعي وحيوي وبيئي يركز على المجتمع.

الخلاصة: لدى الإدارة الكاملة لناقلات الأمراض تأثير في تنقيص أماكن تكاثر الجراثيم ومؤشر الحشرات ولكن الدليل   على التأثيرات على النتائح الصحية محدود. وكانت النتائج الإجتماعية عبارةً عن قدرات وإمكانيات محسنة، وتمكين، ومعرفة مجتمعية. كانت التقويمات الاقتصادية نادرةً، وكانت الفعالية من حيث التكلفة معتمدةً على انتشار المرض. يعد بناء قدرات المجتمعات المحلية المكون الرئيس للاستدامة، مع التعاون وإضفاء الطابع المؤسسي وجعل النشاطات روتينيةً. تشير النتائج إلى تنوع كبير في التدخلات، وتسلط الضوء على الحاجة إلى تمييز التدخلات بشّدة لتسهيل قابيلية التحويل.

Translated from English version into Arabic by GHANIA Khalifa, proofread by Amjad Basheer, through

TRANSLATORS WITHOUT BORDERS

城市环境中预防媒介传播疾病方法——媒介生物综合管理的影响因素、经济评价和可持续性评估：勘域综述

Jorge Marcos-Marcos' Antonio Olry de Labry-Lima, Silvia Toro-Cardenas, Marina Lacasaña, Stéphanie Degroote, Valéry Ridde, Clara Bermudez-Tamayo

摘要

引言：媒介传播疾病（VBD）防治是全球卫生议程面临的最大挑战之一。快速、不受控制的城市化进程，提高了研究者通过媒介生物综合管理（IVM）方法解决这些挑战的关注度。其目的是确定与影响因素、经济评价和可持续性相关的因素，这些因素可能是有助于预防 VBD 的综合性措施。

主要内容：我们在 Pubmed、Web of Science、CINAHL、Econlit、LILACS、全球健康数据库、Scopus 和 Embase 以及热带病公告、WHOLIS、WHO 农药评估计划、Google 学术搜索等数据库，应用 MeSH 术语和自由文本术语对现有文献（2000–2016）进行检索。使用数据提取表格，包括 TIDieR 和 ASTARE 提取数据。应用 MMAT 和 CHEERS 进行质量评估。
在审查的 42 份文献中，有 30 份关注登革热，8 份涉及疟疾，2 份为利什曼病。超过半数的研究在美洲进行。其中半数采用定量描述性方法（$n = 21$），随后进行整群随机对照试验（$n = 11$）。影响因素包括：a）使用媒介控制措施，b）媒介控制，c）卫生措施，和 d）社会措施。IVM 减少了孳生地，降低了昆虫学指数（entomology index）和寄生虫率（parasite rates）。IVM 效果出现异质性，变化幅度很大，但在所有情况下均有利于干预。IVM 对健康结局影响的证据非常有限，但显示发病率降低。社会成果包括才能和能力、赋权和社区知识的提升。关于经济评估，仅四项研究进行了经济分析，干预效果超过成本。成本效益取决于疾病发生率。相关研究从三个维度（社会、经济和环境）来分析可持续性的关键要素，强调实施以社区为重点的生态-生物-社会学方法。
结论：IVM 可减少媒介孳生地和降低昆虫学指数，但对健康结果影响的证据有限。社会成果包括才能和能力、赋权和社区知识的提升。关于经济评估则很少涉及，成本效益取决于疾病发生率。社区能力建设、合作化、制度化和活动日常化是可持续发展的重要组成部分。调查结果显示干预措施实施效果之间存在较大的异质性，并强调严格确定干预措施的特征，以促进其可转让性。

Translated from English version into Chinese by Xue-Jiao Teng, edited by Pin Yang

Impact, évaluation économique et durabilité de la gestion intégrée des vecteurs en milieu urbain pour prévenir les maladies à transmission vectorielle : une étude sur les possibilités

Jorge Marcos-Marcos, Antonio Olry de Labry-Lima, Silvia Toro-Cardenas, Marina Lacasaña, Stéphanie Degroote, Valéry Ridde, Clara Bermudez-Tamayo

Résumé

Contexte: La lutte contre les maladies à transmission vectorielle (MVBD) est l'un des plus grands défis de l'agenda sanitaire mondial. L'urbanisation rapide et incontrôlée a accru l'intérêt à relever ces défis par le biais d'une approche de gestion intégrée des vecteurs (GIV). L'objectif était d'identifier les composantes liées aux impacts et à l'évaluation économique, et à la durabilité qui pourraient contribuer à cette approche intégrée de la prévention de la MVB.
Corps principal: Nous avons effectué une revue littéraire (2000-2016) disponible via PubMed, Web of Science, Cochrane, CINAHL, Econlit, LILACS, Global Health Database, Scopus et Embase, ainsi que le Bulletin des maladies tropicales, WHOLIS, le Système d'évaluation des pesticides de l'OMS et Google Scholar. Les termes MeSH et les termes en texte libre ont été utilisés. Un formulaire d'extraction de données a été utilisé, y compris TIDieR et ASTAIRE. MMAT et CHEERS pour évaluer la qualité. Sur les 42 documents examinés, 30 portaient sur la dengue, huit sur le paludisme et deux sur la leishmaniose. Plus de la moitié des études ont été menées dans les Amériques. La moitié de ces études ont utilisé une approche descriptive quantitative ($n = 21$), suivie d'essais contrôlés randomisés en grappes ($n = 11$). En ce qui concerne les impacts, le bilan a été : a) l'utilisation de mesures de lutte antivectorielle ; b) la lutte antivectorielle ; c) les mesures sanitaires ; et d) les mesures sociales. La MIV a réduit les sites de reproduction, l'indice entomologique et les taux de parasites. Les résultats étaient hétérogènes, avec des amplitudes variables, mais dans tous les cas favorables à l'intervention. Les preuves MIV d'impacts sur les résultats en terme de santé étaient très limitées mais ont montré une

incidence réduite. Les résultats sociaux ont été l'amélioration des aptitudes et des capacités, l'autonomisation et une meilleure connaissance de la communauté. En ce qui concerne l'évaluation économique, seules quatre études ont effectué une analyse économique et les avantages de l'intervention l'emportent sur les coûts. Le rapport coût-efficacité dépendait de l'incidence de la maladie. Les résultats ont fourni des éléments clés pour analyser la durabilité en termes de trois dimensions (sociale, économique et environnementale), en mettant l'accent sur la mise en œuvre d'une approche éco-bio-sociale axée sur la communauté.

Conclusions: La MIV a un impact sur la réduction des sites de reproduction des vecteurs et de l'indice entomologique, mais les preuves de ces impacts sur les résultats pour la santé sont limitées. Les résultats sociaux sont l'amélioration des capacités, l'autonomisation et le savoir sur la communauté. Les évaluations économiques sont rares, et le rapport coût-efficacité dépend de l'incidence de la maladie. Le renforcement des capacités communautaires est la principale composante de la durabilité, avec la collaboration, l'institutionnalisation et la routinisation des activités. Les résultats indiquent une grande hétérogénéité dans les interventions et soulignent la nécessité de caractériser rigoureusement les interventions pour en faciliter la transférabilité.

Translated from English version into French by AiLe_, through

Воздействие, экономическая оценка и долговременность комплексной борьбы с переносчиками болезнетворных организмов в городских условиях для предотвращения трансмиссивных болезней: обзор аналитических исследований

Хорхе Маркос-Маркос’ Антонио Олри де Лабри-Лима, Сильвия Торо-Карденас, Марина Лакасьяна, Стефани Дегрут, Валери Ридд, Клара Бермудес-Тамайо

Аннотация

Общие сведения: Контроль трансмиссивных болезней является одной из самых серьезных проблем глобальной повестки дня в области здравоохранения. Быстрая и неконтролируемая урбанизация усилила интерес к решению этих проблем с помощью комплексной борьбы с переносчиками болезнетворных организмов. Цель заключалась в выявлении компонентов, связанных с воздействием, экономической оценкой и долговременностью, которые могли бы внести вклад в этот комплексный подход к профилактике трансмиссивных болезней.

Основная часть: Мы провели предварительный обзор доступной литературы (2000-2016 гг.) с использованием таких источников, как PubMed, Web of Science, Cochrane, CINAHL, Econlit, LILACS, Глобальной базы данных по здравоохранению, Scopus и Embase, а также бюллетеня по тропическим болезням, WHOLIS, схемы оценки пестицидов WHO и Google Scholar. Были использованы термины MeSH и термины свободного текстового поиска. Использовалась форма извлечения данных, включая TIDieR и ASTAIRE. Для оценки качества применялись MMAT и CHEERS. Из 42 рассмотренных документов 30 были сосредоточены на лихорадке денге, восемь – на малярии, и два – на лейшманиозе. Более половины исследований были проведены в Северной и Южной Америке. В половине был использован количественный описательный подход (n = 21), а затем кластерные рандомизированные контролируемые исследования (n = 11). Что касается воздействия, то результаты

были следующими: а) использование мероприятий для векторного контроля; б) векторный контроль; с) мероприятия в области здравоохранения; и d) социальные мероприятия. Комплексная борьба с переносчиками болезнетворных организмов сократила места размножения, индекс энтомологии и паразитарный индекс. Результаты были неоднородными с переменными величинами, но во всех случаях благоприятными для вмешательства. Доказательства воздействия комплексной борьбы с переносчиками болезнетворных организмов на показатели состояния здоровья были очень ограниченными, но показали снижение частоты заболеваний. Социальными результатами стали улучшенные способностей и возможностей, расширение прав и знаний сообщества. Что касается экономической оценки, то только четыре исследования провели экономический анализ, и польза от вмешательства перевесила издержки. Рентабельность зависела от частоты заболеваний. Результаты обеспечили ключевыми элементами для анализа долговременности в трех измерениях (социальном, экономическом и экологическом), в которых особое внимание уделялось внедрению эко-биосоциального подхода, ориентированного на общины.

Итоги: Комплексная борьба с переносчиками болезнетворных организмов оказывает влияние на сокращение участков размножения и индекса энтомологии, но данные о влиянии на показатели состояния здоровья ограничены. Социальными результатами являются улучшенные возможности и потенциял, расширение прав и знаний в сообществах. Экономические оценки недостаточны, а экономическая эффективность зависит от частоты заболеваний. Создание потенциала сообщества является основным компонентом долговременности, а также сотрудничества, институционализации и проведения повторных мероприятий. Выводы указывают на большую неоднородность вмешательств и подчеркивают необходимость тщательного описания вмешательств, чтобы облегчить применимость информации.

Translated from English version into Russian by Oksana Rozhko, proofread by Liudmila Tomanek, through

Impacto, evaluación económica y sostenibilidad del manejo integrado de portadores en entornos urbanos para prevenir enfermedades transmitidas por portadores: una revisión del alcance

Jorge Marcos-Marcos, Antonio Olry de Labry-Lima, Silvia Toro-Cardenas, Marina Lacasaña, Stéphanie Degroote, Valéry Ridde, Clara Bermudez-Tamayo

Reseña

Antecedentes: El control de las enfermedades transmitidas por portadores (ETP) es uno de los mayores desafíos en la agenda de salud global. La urbanización rápida e incontrolada ha aumentado el interés en abordar estos desafíos mediante un enfoque de gestión integrada de portadores (GIP). El objetivo fue identificar los componentes relacionados con los impactos, la evaluación económica y la sostenibilidad que podrían contribuir a este enfoque integrado para la prevención de ETP.

Cuerpo principal: Realizamos una revisión del alcance de la literatura disponible (2000-2016) utilizando PubMed, Web of Science, Cochrane, CINAHL, Econlit, LILACS, Global Health Database, Scopus y Embase, así como Boletín de Enfermedades Tropicales, WHOLIS, Esquema de Evaluación de Pesticidas de la OMS y Google Scholar. Se usaron

términos MeSH y términos de texto libre. Se utilizó un formulario de extracción de datos, que incluye TIDieR y ASTAIRE. MMAT y CHEERS para evaluar la calidad. De los 42 documentos revisados, 30 se centraron en dengue, ocho en malaria y dos en leishmaniosis. Más de la mitad de los estudios se realizaron en las Américas. La mitad utilizó un enfoque descriptivo cuantitativo (*n* = 21), seguidos por ensayos controlados aleatorios de grupos temáticos (*n* = 11). En cuanto a los impactos, los resultados fueron: a) uso de medidas para el control de portadores; b) control de portadores; c) medidas de salud; y d) medidas sociales. GIP redujo los sitios de reproducción, el índice de entomología y las tasas de parásitos. Los resultados fueron heterogéneos, con magnitudes variables, pero en todos los casos fueron favorables a la intervención. Evidencia de los impactos de GIP en los resultados de salud fue muy limitada, pero mostró una incidencia reducida. Los resultados sociales fueron habilidades y capacidades mejoradas, empoderamiento y conocimiento de la comunidad. Con respecto a la evaluación económica, solo cuatro estudios realizaron un análisis económico, y los beneficios de intervención superaron los costes. La relación coste-efectividad fue dependiente de la incidencia de la enfermedad. Los resultados proporcionaron elementos clave para analizar la sostenibilidad en términos de tres dimensiones (social, económico y ambiental), enfatizando la implementación de un enfoque eco-bio-social centrado en la comunidad.

Conclusiones: El GIP tiene un impacto en la reducción de los puntos de reproducción de los vectores y el indice de entomología, pero la evidencia de los impactos en los resultados de salud es limitada. Los resultados sociales son habilidades y capacidades mejoradas, empoderamiento y conocimiento de la comunidad. Las evaluaciones económicas son escasas, y la relación coste-efectividad depende de la incidencia de la enfermedad. El desarrollo de la capacidad comunitaria es el componente principal de la sostenibilidad, junto con la colaboración, la institucionalización y la rutina de actividades. Los resultados indican una gran heterogeneidad en las intervenciones y resaltan la necesidad de caracterizar las intervenciones rigurosamente para facilitar la transferibilidad.

Translated from English version into Spanish by Ignacio Montalvo, proofread by Andrea Barrocal Velasco, through

TRANSLATORS
WITHOUT BORDERS
