# Supplementary material for: Impact, economic evaluation, and sustainability of integrated vector management in urban settings to prevent vector-borne diseases: a scoping review
Source: Infect Dis Poverty. 2018 Sep 3;7:83. doi: 10.1186/s40249-018-0464-x (PMC6120095; doi:10.1186/s40249-018-0464-x)
Supplement: Supplementary file 2 — Complete search strategy by database. (DOCX 31 kb) [file 40249_2018_464_MOESM2_ESM.docx]

**Additional file 1: COMPLETE SEARCH STRATEGY BY DATABASE**

Search for relevant studies in **Medline (PubMed)** Database:

| **Keywords / Descriptors** |
| --- |
| Search (((((((((Program Evaluation[MeSH Terms] OR Costs and Cost Analysis[MeSH Terms] OR Evaluation studies[MeSH Terms] OR Efficiency[MeSH Terms]))) OR (("Program Evaluation"[Title/Abstract] OR "Health Program"[Title/Abstract] OR "Evaluation Program"[Title/Abstract] OR "Evaluation Project"[Title/Abstract] OR "Project Evaluation"[Title/Abstract] OR "Social Validity Research"[Title/Abstract] OR "Impact analysis"[Title/Abstract] OR "cost-effectiveness"[Title/Abstract] OR "cost effectiveness"[Title/Abstract] OR “cost-benefit”[Title/Abstract] OR “cost-efficiency”[Title/Abstract] OR sustainab*[Title/Abstract] OR institutionalization[Title/Abstract] OR routinization[Title/Abstract] OR "impact assessment"[Title/Abstract] OR "Impact Evaluation"[Title/Abstract] OR “Program* effect*”[Title/Abstract] OR “Program* outcome*"[Title/Abstract])))) AND ((((Pest control[MeSH Terms] OR Insect control[MeSH Terms] OR Mosquito control[MeSH Terms] OR Communicable Disease Control[MeSH Terms] OR Tick control[MeSH Terms] OR Infection control[MeSH Terms] OR Biological Control Agents[MeSH Terms]))) OR (("Integrated Vector Management (IVM)"[Title/Abstract] OR “Vector* control environmental management”[Title/Abstract] OR "Vector Management”[Title/Abstract] OR “Integrated Pest Management (IPM)”[Title/Abstract] OR "vector control"[Title/Abstract] OR "control program*”[Title/Abstract] OR "pest control"[Title/Abstract] OR “Insect control”[Title/Abstract] OR “Mosquito control”[Title/Abstract] OR “Tick control”[Title/Abstract] OR “Communicable Disease Control”[Title/Abstract] OR “Infection control”[Title/Abstract] OR “Biological Control Agents”[Title/Abstract] OR “long lasting insecticidal nets (LLINs)”[Title/Abstract] OR “larval source management (LSM)”[Title/Abstract] OR “indoor residual spraying (IRS)”[Title/Abstract] OR “chemical control”[Title/Abstract] OR “biological control”[Title/Abstract] OR “Habitat control”[Title/Abstract] OR “Environmental control”[Title/Abstract])))) AND (((((Disease Transmission, Infectious[MeSH Terms] OR Disease Vectors[MeSH Terms] OR Communicable Diseases[MeSH Terms] OR Communicable Diseases, Emerging[MeSH Terms] OR Neglected Diseases[MeSH Terms] OR Insect Vectors[MeSH Terms] OR Arthropod Vectors[MeSH Terms] OR Arachnid Vectors[MeSH Terms]))) OR ((Sandfly fever Naples virus[MeSH Terms] OR phlebovirus[MeSH Terms] OR phlebotomus fever[MeSH Terms] OR Arthropod vectors[MeSH Terms]OR Schistosomiasis[MeSH Terms] OR filariasis[MeSH Terms] OR Chikungunya virus[MeSH Terms] OR chikungunya fever[MeSH Terms] OR Leishmaniasis[MeSH Terms] OR Leishmaniasis, Visceral[MeSH Terms] OR Hemorrhagic Fever Virus[MeSH Terms] OR Hemorrhagic Fever, Crimean[MeSH Terms] OR Lyme disease[MeSH Terms] OR Relapsing fever[MeSH Terms] OR Borrelia Infections[MeSH Terms] OR Rickettsia[MeSH Terms] OR Rickettsia Infections[MeSH Terms] OR Tick-Borne infections[MeSH Terms] OR Q Fever[MeSH Terms] OR Coxiella burneti[MeSH Terms] OR Encephalitis Viruses, Tick-Borne Encephalitis, Tick-Borne[MeSH Terms] OR Tularaemia[MeSH Terms] OR Chagas disease[MeSH Terms] OR Trypanosomiasis, African[MeSH Terms] OR Malaria[MeSH Terms] OR Dengue[MeSH Terms] OR Rift valley fever[MeSH Terms] OR Rift valley fever virus[MeSH Terms] OR Yellow fever[MeSH Terms] OR Zika virus infection[MeSH Terms] OR Zika virus[MeSH Terms] OR Encephalitis, Japanese[MeSH Terms] OR Elephantiasis, Filarial[MeSH Terms] OR West Nile fever[MeSH Terms] OR Schistosomiasis[MeSH Terms] OR Trypanosomiasis, African[MeSH Terms] OR Plague[MeSH Terms] OR Yersinia pestis[MeSH Terms] OR Rickettsia[MeSH Terms] OR Rickettsia Infections[MeSH Terms] OR Onchocerciasis[MeSH Terms] OR Onchocerciasis, Ocular[MeSH Terms] OR Aedes[MeSH Terms] OR Dog diseases[MeSH Terms]))) OR (("Vector-borne disease*"[Title/Abstract] OR "mosquito-borne disease*"[Title/Abstract] OR "mosquito disease*"[Title/Abstract] OR "zoonotic disease*"[Title/Abstract] OR "zoonotic pathogen*"[Title/Abstract] OR "vector-borne pathogen*"[Title/Abstract] OR "Insect Vector*"[Title/Abstract] OR "Emerging disease*"[Title/Abstract] OR "Re-Emerging disease*"[Title/Abstract] OR "Arthropod-borne virus*"[Title/Abstract] OR "arthropodborne disease*"[Title/Abstract] OR "arthropod-transmitted zoonose*"[Title/Abstract] OR "communicable disease"[Title/Abstract] OR "neglected disease*"[Title/Abstract] OR "tropical disease*"[Title/Abstract] OR Arbovirus*[Title/Abstract] OR Flaviviruse*[Title/Abstract] OR Dengue*[Title/Abstract] OR Zika*[Title/Abstract] OR "West Nile Virus*"[Title/Abstract] OR "west Nile fever"[Title/Abstract] OR "bovine viral diarrhea virus"[Title/Abstract] OR BVDV[Title/Abstract] OR "bovine viral diarrhoea"[Title/Abstract] OR "bovine virus diarrhoea"[Title/Abstract] OR "Japanese Encephalitis"[Title/Abstract] OR "Yellow Fever*"[Title/Abstract] OR Alphaviruse*[Title/Abstract] OR Chikungunya*[Title/Abstract] OR "encephalitis virus*"[Title/Abstract] OR "paralytic virus*"[Title/Abstract] OR malaria*[Title/Abstract] OR filariasis[Title/Abstract] OR leishmaniasis[Title/Abstract] OR trypanosome*[Title/Abstract] OR "parasitic disease*"[Title/Abstract] OR "bacterial disease*"[Title/Abstract] OR Bartonellosis[Title/Abstract] OR plague*[Title/Abstract] OR tularaemia*[Title/Abstract] OR typhus[Title/Abstract] OR "vector-borne zoonose"[Title/Abstract] OR "rift valley fever*"[Title/Abstract] OR "sandfly fever*"[Title/Abstract] OR "phelebotomus fever"[Title/Abstract] OR "Chagas disease*"[Title/Abstract] OR "American tripanosomiasis"[Title/Abstract] OR "Sleeping sickness"[Title/Abstract] OR "African trypanosomiasis"[Title/Abstract] OR Rickettsiosis[Title/Abstract] OR Onchocerciasis[Title/Abstract] OR "river blindness"[Title/Abstract] OR Schistosomiasis[Title/Abstract] OR bilharziasis[Title/Abstract] OR "Relapsing fever*"[Title/Abstract] OR borreliosis[Title/Abstract] OR "Rickettsial disease*"[Title/Abstract] OR "spotted fever"[Title/Abstract] OR "Q fever"[Title/Abstract] OR "Tick-borne encephalitis"[Title/Abstract] OR Tularaemia*[Title/Abstract] OR "Crimean-Congo haemorrhagic fever*"[Title/Abstract] OR "Lyme disease*"[Title/Abstract] OR Rabie*[Title/Abstract] OR "dog disease*"[Title/Abstract])))) AND ((((Urban Population[MeSH Terms] OR Urban health[MeSH Terms] OR Cities[MeSH Terms] OR urbanization[MeSH Terms]))) OR ((urban*[Title/Abstract] OR “urban area*”[Title/Abstract] OR “urban population*”[Title/Abstract] OR “urban spatial”[Title/Abstract] OR “urban setting”[Title/Abstract] OR “urban environment*”[Title/Abstract] “urban ecosystem*”[Title/Abstract] OR municipal*[Title/Abstract] OR cities[Title/Abstract] OR city[Title/Abstract] OR "Large Cities”[Title/Abstract] OR "Medium Cities"[Title/Abstract] OR "Small Cities"[Title/Abstract] OR neighborhood*[Title/Abstract] OR neighbourhood*[Title/Abstract] OR town*[Title/Abstract] OR slum*[Title/Abstract] OR district*[Title/Abstract] OR suburb*[Title/Abstract]))))) |

Search for relevant studies in **Web of Science** Database:

| **Keywords / Descriptors** |
| --- |
| ("Program Evaluation" OR "intervention evaluation" OR "Costs and Cost Analysis" OR "Evaluation stud*" OR efficiency OR "Health Program" OR "Evaluation Program" OR "Evaluation Project" OR "Project Evaluation" OR "Social Validity Research" OR "Impact analysis" OR "cost-effectiveness" OR "cost effectiveness" OR "cost-benefit" OR "cost-efficiency" OR sustainab* OR institutionalization OR routinization OR "impact assessment" OR "Impact Evaluation" OR "Program* effect*" OR "Program* outcome*") AND ("Pest control" OR "Insect control" OR "Mosquito control" OR "Communicable Disease Control" OR "Tick control" OR "Infection control" OR "Biological Control Agents" OR "Integrated Vector Management" OR "Vector* control environmental management" OR "Vector Management" OR "Integrated Pest Management" OR "vector control" OR "control program*" OR "long lasting insecticidal nets" OR "larval source management" OR "indoor residual spraying" OR "chemical control" OR "biological control" OR "Habitat control" OR "Environmental control") AND ("Disease Transmission" OR "Disease Vector*" OR "Communicable Disease*" OR "Neglected Disease*" OR "tropical disease*" OR "Insect Vector*" OR "Arthropod Vector*" OR "Arachnid Vector*" OR "Sandfly fever Naples virus" OR phlebovirus OR "phlebotomus fever" OR schistosomiasis OR filariasis OR "Chikungunya virus" OR "chikungunya fever" OR leishmaniasis OR "Hemorrhagic Fever Virus" OR "Crimean-Congo Hemorrhagic Fever" OR "Lyme disease*" OR "Relapsing fever" OR "Borrelia Infections" OR rickettsia OR "Rickettsia Infections" OR "Tick-Borne infections" OR "Q Fever" OR "Coxiella burneti" OR "Tick-Borne Encephalitis" OR tick-borne OR "Chagas disease" OR "African Trypanosomiasis" OR malaria OR dengue OR "Rift valley fever" OR "Rift valley fever virus" OR "Yellow fever" OR "Zika virus infection" OR "Zika virus" OR "Japanese Encephalitis" OR elephantiasis OR "West Nile fever" OR schistosomiasis OR "African Trypanosomiasis" OR plague OR "Yersinia pestis" OR rickettsia OR "Rickettsia Infections" OR onchocerciasis OR aedes OR "Dog disease*" OR "Vector-borne disease*" OR "mosquito-borne disease*" OR "mosquito disease*" OR "zoonotic disease*" OR "zoonotic pathogen*" OR "vector-borne pathogen*" OR "Emerging disease*" OR "Re-Emerging disease*" OR "Arthropod-borne virus*" OR "arthropodborne disease*" OR "arthropod-transmitted zoonose*" OR arbovirus* OR flaviviruse* OR dengue* OR zika* OR "bovine viral diarrhea virus" OR "bovine viral diarrhoea" OR alphaviruse* OR chikungunya* OR "encephalitis virus*" OR "paralytic virus*" OR trypanosome* OR "parasitic disease*" OR "bacterial disease*" OR bartonellosis OR plague* OR tularaemia* OR typhus OR "vector-borne zoonose" OR "spotted fever" OR rabie*) AND ("Urban Population" OR "Urban health" OR cities OR city OR urbanization OR urban* OR "urban area*" OR "urban spatial" OR "urban setting" OR "urban environment*" OR "urban ecosystem*" OR municipal* OR "Large Cities" OR "Medium Cities" OR "Small Cities" OR neighborhood* OR neighbourhood* OR town* OR slum* OR district* OR suburb*) |

Search for relevant studies in **Cochrane Library** Database:

| **Keywords / Descriptors** |
| --- |
| Program Evaluation" OR "intervention evaluation" OR "Costs and Cost Analysis" OR "Evaluation stud*" OR efficiency OR "Health Program" OR "Evaluation Program" OR "Evaluation Project" OR "Project Evaluation" OR "Social Validity Research" OR "Impact analysis" OR "cost-effectiveness" OR "cost effectiveness" OR "cost-benefit" OR "cost-efficiency" OR sustainab* OR institutionalization OR routinization OR "impact assessment" OR "Impact Evaluation" OR "Program* effect*" OR "Program* outcome*" in Title, Abstract, Keywords **and** "Pest control" OR "Insect control" OR "Mosquito control" OR "Communicable Disease Control" OR "Tick control" OR "Infection control" OR "Biological Control Agents" OR "Integrated Vector Management" OR "Vector* control environmental management" OR "Vector Management" OR "Integrated Pest Management" OR "vector control" OR "control program*" OR "long lasting insecticidal nets" OR "larval source management" OR "indoor residual spraying" OR "chemical control" OR "biological control" OR "Habitat control" OR "Environmental control" in Title, Abstract, Keywords **and** "Disease Transmission" OR "Disease Vector" OR "Disease vectors" OR "Communicable Disease" OR "Communicable Diseases" OR "Neglected Disease" OR "Neglected Diseases "OR "tropical disease" OR "tropical diseases" OR "Insect Vector" OR "Insect Vectors" OR "Arthropod Vector" OR "Arthropod Vectors" OR "Arachnid Vector" OR "Arachnid Vectors" OR "Sandfly fever Naples virus" OR phlebovirus OR "phlebotomus fever" OR schistosomiasis OR filariasis OR "Chikungunya virus" OR "chikungunya fever" OR leishmaniasis OR "Hemorrhagic Fever Virus" OR "Crimean-Congo Hemorrhagic Fever" OR "Lyme disease" OR "Lyme diseases" OR "Relapsing fever" OR "Borrelia Infections" OR rickettsia OR "Rickettsia Infections" OR "Tick-Borne infections" OR "Q Fever" OR "Coxiella burneti" OR "Tick-Borne Encephalitis" OR tick-borne OR tularaemia* OR "Chagas disease" OR "African Trypanosomiasis" OR malaria OR dengue OR "Rift valley fever" OR "Rift valley fever virus" OR "Yellow fever" OR "Zika virus infection" OR "Zika virus" OR "Japanese Encephalitis" OR elephantiasis OR "West Nile fever" OR schistosomiasis OR "African Trypanosomiasis" OR plague OR "Yersinia pestis" OR rickettsia OR "Rickettsia Infections" OR onchocerciasis OR aedes OR "Dog disease" OR "Dog diseases" OR "Vector-borne disease" OR "Vector-borne diseases" OR "mosquito-borne disease" OR "mosquito-borne diseases" OR "mosquito disease" OR "mosquito diseases" OR "zoonotic disease" OR "zoonotic diseases" OR "zoonotic pathogen" OR "zoonotic pathogens" OR "vector-borne pathogen" OR "vector-borne pathogens" OR "Emerging disease" OR "Emerging diseases" OR "Re-Emerging disease" OR "Re-Emerging diseases" OR "Arthropod-borne virus" OR "arthropodborne disease" OR "arthropodborne diseases" OR "arthropod-transmitted zoonose" OR "arthropod-transmitted zoonoses" OR arbovirus OR flavivirus OR dengue OR zika OR "bovine viral diarrhea virus" OR "bovine viral diarrhoea" OR alphavirus OR chikungunya OR "encephalitis virus" OR "paralytic virus" OR trypanosoma OR trypanosomes OR "parasitic disease" OR "parasitic diseases" OR "bacterial disease" OR "bacterial diseases" OR bartonellosis OR plague OR typhus OR "vector-borne zoonose" OR "spotted fever" OR rabie* in Title, Abstract, Keywords **and** "Urban Population" OR "Urban health" OR cities OR city OR urbanization OR urban* OR "urban area" OR "urban areas" OR "urban spatial" OR "urban setting" OR "urban environment" OR "urban ecosystem" OR municipal* OR "Large Cities" OR "Medium Cities" OR "Small Cities" OR neighborhood* OR neighbourhood* OR town* OR slum* OR district* OR suburb* in Title, Abstract, Keywords |

Search for relevant studies in **CINAHL Complete** Database:

| **Keywords / Descriptors** |
| --- |
| AB ( "Program Evaluation" OR "intervention evaluation" OR "Costs and Cost Analysis" OR "Evaluation stud*" OR efficiency OR "Health Program" OR "Evaluation Program" OR "Evaluation Project" OR "Project Evaluation" OR "Social Validity Research" OR "Impact analysis" OR "cost-effectiveness" OR "cost effectiveness" OR "cost-benefit" OR "cost-efficiency" OR sustainab* OR institutionalization OR routinization OR "impact assessment" OR "Impact Evaluation" OR "Program* effect*" OR "Program* outcome*" ) AND AB ( "Pest control" OR "Insect control" OR "Mosquito control" OR "Communicable Disease Control" OR "Tick control" OR "Infection control" OR "Biological Control Agents" OR "Integrated Vector Management" OR "Vector* control environmental management" OR "Vector Management" OR "Integrated Pest Management" OR "vector control" OR "control program*" OR "long lasting insecticidal nets" OR "larval source management" OR "indoor residual spraying" OR "chemical control" OR "biological control" OR "Habitat control" OR "Environmental control" ) AND AB ( "Disease Transmission" OR "Disease Vector" OR "Disease vectors" OR "Communicable Disease" OR "Communicable Diseases" OR "Neglected Disease" OR "Neglected Diseases "OR "tropical disease" OR "tropical diseases" OR "Insect Vector" OR "Insect Vectors" OR "Arthropod Vector" OR "Arthropod Vectors" OR "Arachnid Vector" OR "Arachnid Vectors" OR "Sandfly fever Naples virus" OR phlebovirus OR "phlebotomus fever" OR schistosomiasis OR filariasis OR "Chikungunya virus" OR "chikungunya fever" OR leishmaniasis OR "Hemorrhagic Fever Virus" OR "Crimean-Congo Hemorrhagic Fever" OR "Lyme disease" OR "Lyme diseases" OR "Relapsing fever" OR "Borrelia Infections" OR rickettsia OR "Rickettsia Infections" OR "Tick-Borne infections" OR "Q Fever" OR "Coxiella burneti" OR "Tick-Borne Encephalitis" OR tick-borne OR tularaemia* OR "Chagas disease" OR "African Trypanosomiasis" OR malaria OR dengue OR "Rift valley fever" OR "Rift valley fever virus" OR "Yellow fever" OR "Zika virus infection" OR "Zika virus" OR "Japanese Encephalitis" OR elephantiasis OR "West Nile fever" OR schistosomiasis OR "African Trypanosomiasis" OR plague OR "Yersinia pestis" OR rickettsia OR "Rickettsia Infections" OR onchocerciasis OR aedes OR "Dog disease" OR "Dog diseases" OR "Vector-borne disease" OR "Vector-borne diseases" OR "mosquito-borne disease" OR "mosquito-borne diseases" OR "mosquito disease" OR "mosquito diseases" OR "zoonotic disease" OR "zoonotic diseases" OR "zoonotic pathogen" OR "zoonotic pathogens" OR "vector-borne pathogen" OR "vector-borne pathogens" OR "Emerging disease" OR "Emerging diseases" OR "Re-Emerging disease" OR "Re-Emerging diseases" OR "Arthropod-borne virus" OR "arthropodborne disease" OR "arthropodborne diseases" OR "arthropod-transmitted zoonose" OR "arthropod-transmitted zoonoses" OR arbovirus OR flavivirus OR dengue OR zika OR "bovine viral diarrhea virus" OR "bovine viral diarrhoea" OR alphavirus OR chikungunya OR "encephalitis virus" OR "paralytic virus" OR trypanosoma OR trypanosomes OR "parasitic disease" OR "parasitic diseases" OR "bacterial disease" OR "bacterial diseases" OR bartonellosis OR plague OR typhus OR "vector-borne zoonose" OR "spotted fever" OR rabie* ) AND AB ( "Urban Population" OR "Urban health" OR cities OR city OR urbanization OR urban* OR "urban area" OR "urban areas" OR "urban spatial" OR "urban setting" OR "urban environment" OR "urban ecosystem" OR municipal* OR "Large Cities" OR "Medium Cities" OR "Small Cities" OR neighborhood* OR neighbourhood* OR town* OR slum* OR district* OR suburb* ) |

Search for relevant studies in **Econlit** Database:

| **Keywords / Descriptors** |
| --- |
| AB ( "Program Evaluation" OR "intervention evaluation" OR "Costs and Cost Analysis" OR "Economic evaluation" OR "Evaluation stud*" OR efficiency OR "Health Program" OR "Evaluation Program" OR "Evaluation Project" OR "Project Evaluation" OR "Social Validity Research" OR "Impact analysis" OR "cost-effectiveness" OR "cost effectiveness" OR "cost-benefit" OR "cost-efficiency" OR “Cost-Effectiveness of Control” OR sustainab* OR institutionalization OR routinization OR "impact assessment" OR "Impact Evaluation" OR "Program* effect*" OR "Program* outcome*" ) AND AB ( "Pest control" OR "Insect control" OR "Mosquito control" OR "Communicable Disease Control" OR "Tick control" OR "Infection control" OR "Biological Control Agents" OR "Integrated Vector Management" OR "Vector* control environmental management" OR "Vector Management" OR "Integrated Pest Management" OR "vector control" OR "control program*" OR "long lasting insecticidal nets" OR "larval source management" OR "indoor residual spraying" OR "chemical control" OR "biological control" OR "Habitat control" OR "Environmental control" ) AND AB ( "Disease Transmission" OR "Disease Vector*" OR "Communicable Disease*" OR "Neglected Disease*" OR "tropical disease*" OR “Infectious Vector Diseases” OR "Insect Vector*" OR "Arthropod Vector*" OR "Arachnid Vector*" OR "Sandfly fever Naples virus" OR phlebovirus OR "phlebotomus fever" OR schistosomiasis OR filariasis OR "Chikungunya virus" OR "chikungunya fever" OR leishmaniasis OR "Hemorrhagic Fever Virus" OR "Crimean-Congo Hemorrhagic Fever" OR "Lyme disease*" OR "Relapsing fever" OR "Borrelia Infections" OR rickettsia OR "Rickettsia Infections" OR "Tick-Borne infections" OR "Q Fever" OR "Coxiella burneti" OR "Tick-Borne Encephalitis" OR tick-borne OR tularaemia* OR "Chagas disease" OR "African Trypanosomiasis" OR malaria OR dengue OR "Rift valley fever" OR "Rift valley fever virus" OR "Yellow fever" OR "Zika virus infection" OR "Zika virus" OR "Japanese Encephalitis" OR elephantiasis OR "West Nile fever" OR schistosomiasis OR "African Trypanosomiasis" OR plague OR "Yersinia pestis" OR rickettsia OR "Rickettsia Infections" OR onchocerciasis OR aedes OR "Dog disease*" OR "Vector-borne disease*" OR "mosquito-borne disease*" OR "mosquito disease*" OR "zoonotic disease*" OR "zoonotic pathogen*" OR "vector-borne pathogen*" OR "Emerging disease*" OR "Re-Emerging disease*" OR "Arthropod-borne virus*" OR "arthropodborne disease*" OR "arthropod-transmitted zoonose*" OR arbovirus* OR flaviviruse* OR dengue* OR zika* OR "bovine viral diarrhea virus" OR "bovine viral diarrhoea" OR alphaviruse* OR chikungunya* OR "encephalitis virus*" OR "paralytic virus*" OR trypanosome* OR "parasitic disease*" OR "bacterial disease*" OR bartonellosis OR plague* OR tularaemia* OR typhus OR "vector-borne zoonose" OR "spotted fever" OR rabie* ) AND AB ( "Urban Population" OR "Urban health" OR cities OR city OR urbanization OR urban* OR "urban area*" OR "urban spatial" OR "urban setting" OR "urban environment*" OR "urban ecosystem*" OR municipal* OR "Large Cities" OR "Medium Cities" OR "Small Cities" OR neighborhood* OR neighbourhood* OR town* OR slum* OR district* OR suburb* ) |

Search for relevant studies in **Lilacs** Database:

| **Keywords / Descriptors** |
| --- |
| (tw:("Program Evaluation" OR "intervention evaluation" OR "Costs and Cost Analysis" OR "Evaluation study" OR efficiency OR "Health Program" OR "Evaluation Program" OR "Evaluation Project" OR "Project Evaluation" OR "Economic evaluation" OR "Social Validity Research" OR "Impact analysis" OR "cost-effectiveness" OR "cost effectiveness" OR "cost-benefit" OR "cost-efficiency" OR sustainab* OR institutionalization OR routinization OR "impact assessment" OR "Impact Evaluation" OR "Program effectiveness" OR "Program outcome")) AND (tw:("Pest control" OR "Insect control" OR "Mosquito control" OR "Communicable Disease Control" OR "Tick control" OR "Infection control" OR "Biological Control Agents" OR "Integrated Vector Management" OR "Vector* control environmental management" OR "Vector Management" OR "Integrated Pest Management" OR "vector control" OR "control program*" OR "long lasting insecticidal nets" OR "larval source management" OR "indoor residual spraying" OR "chemical control" OR "biological control" OR "Habitat control" OR "Environmental control")) AND (tw:("Disease Transmission" OR "Disease Vector" OR "Disease vectors" OR "Communicable Disease" OR "Communicable Diseases" OR "Neglected Disease" OR "Neglected Diseases "or "tropical disease" OR "tropical diseases" OR "Insect Vector" OR "Insect Vectors" OR "Arthropod Vector" OR "Arthropod Vectors" OR "Arachnid Vector" OR "Arachnid Vectors" OR "Sandfly fever Naples virus" OR phlebovirus OR "phlebotomus fever" OR schistosomiasis OR filariasis OR "Chikungunya virus" OR "chikungunya fever" OR leishmaniasis OR "Hemorrhagic Fever Virus" OR "Crimean-Congo Hemorrhagic Fever" OR "Lyme disease" OR "Lyme diseases" OR "Relapsing fever" OR "Borrelia Infections" OR rickettsia OR "Rickettsia Infections" OR "Tick-Borne infections" OR "Q Fever" OR "Coxiella burneti" OR "Tick-Borne Encephalitis" OR tick-borne OR tularaemia* OR "Chagas disease" OR "African Trypanosomiasis" OR malaria OR dengue OR "Rift valley fever" OR "Rift valley fever virus" OR "Yellow fever" OR "Zika virus infection" OR "Zika virus" OR "Japanese Encephalitis" OR elephantiasis OR "West Nile fever" OR schistosomiasis OR "African Trypanosomiasis" OR plague OR "Yersinia pestis" OR rickettsia OR "Rickettsia Infections" OR onchocerciasis OR aedes OR "Dog disease" OR "Dog diseases" OR "Vector-borne disease" OR "Vector-borne diseases" OR "mosquito-borne disease" OR "mosquito-borne diseases" OR "mosquito disease" OR "mosquito diseases" OR "zoonotic disease" OR "zoonotic diseases" OR "zoonotic pathogen" OR "zoonotic pathogens" OR "vector-borne pathogen" OR "vector-borne pathogens" OR "Emerging disease" OR "Emerging diseases" OR "Re-Emerging disease" OR "Re-Emerging diseases" OR "Arthropod-borne virus" OR "arthropodborne disease" OR "arthropodborne diseases" OR "arthropod-transmitted zoonose" OR "arthropod-transmitted zoonoses" OR arbovirus OR flavivirus OR dengue OR zika OR "bovine viral diarrhea virus" OR "bovine viral diarrhoea" OR alphavirus OR chikungunya OR "encephalitis virus" OR "paralytic virus" OR trypanosoma OR trypanosomes OR "parasitic disease" OR "parasitic diseases" OR "bacterial disease" OR "bacterial diseases" OR bartonellosis OR plague OR typhus OR "vector-borne zoonose" OR "spotted fever" OR rabie*)) AND (tw:("Urban Population" OR "Urban health" OR cities OR city OR urbanization OR urban* OR "urban area" OR "urban areas" OR "urban spatial" OR "urban setting" OR "urban environment" OR "urban ecosystem" OR municipal* OR "Large Cities" OR "Medium Cities" OR "Small Cities" OR neighborhood* OR neighbourhood* OR town* OR slum* OR district* OR suburb*)) |

Search for relevant studies in **Global Health** **Database** by OvidSP (CABS abstracts + Public Health and Tropical Medicine):

| **Keywords / Descriptors** |
| --- |
| exp program evaluation/ or Program Evaluation.mp.  Disease Management.mp. or disease control.sh.  Health Program.mp. or exp health programs/  Project Evaluation.mp. or health programmes.sh. or evaluation.sh.  exp program evaluation/  Disease Management.mp. or disease control.sh. or diagnosis.sh.  Evaluation of Research Programs.mp.  Impact analysis.mp.  cost-effectiveness.mp. or "cost effectiveness analysis".sh. or health care costs.sh.  exp sustainability/ or sustainability.mp.  impact assessment.mp. or assessment.sh. or evaluation.sh.  vector control assessment.mp. or disease control.sh. or vector control.sh.  evaluation of vector control method.mp.  cost-effectiveness of interventions.mp. or "cost benefit analysis".sh.  evidence-based decision-making on vector control.mp.  evaluating the effect of vector control.mp.  Integrated Vector Management.mp. or integrated pest management.sh.  (Vector Ecology and Management).mp. [mp=abstract, title, original title, broad terms, heading words, identifiers, cabicodes]  integrated disease management approach.mp. or integrated control.sh.  new approach to vector control.mp.  (new strategies for prevention and control of vector-borne diseases).mp. [mp=abstract, title, original title, broad terms, heading words, identifiers, cabicodes]  vector-borne disease control programmes.mp.  management approach to vector control.mp.  pest control.mp. or exp pest control/  Disease Transmission, Infectious.mp. or infectious diseases.sh.  Disease Vectors.mp. or exp disease vectors/  Communicable Diseases.mp. or exp infectious diseases/  Neglected Diseases.mp. or tropical diseases.sh.  Insect Vectors.mp. or vector-borne diseases.sh. or vectors.sh. or insect control.sh.  Arthropod Vectors.mp.  Arachnid Vectors.mp.  Infectious Diseases.mp. or exp infectious diseases/  Mosquito control.mp. or vector control.sh. or insect control.sh. or mosquito-borne diseases.sh.  chikungunya.mp. or Chikungunya virus/  dengue fever.mp. or dengue.sh.  rift valley fever.mp. or exp Rift Valley fever/  yellow fever.mp. or exp yellow fever/  zika.mp. or exp Zika virus/  malaria.mp. or exp malaria/  japanese encephalitis.mp. or exp Japanese encephalitis/  lymphatic filariasis.mp. or exp lymphatic filariasis/  west nile fever.mp. or exp West Nile fever/  leishmaniasis.mp. or exp leishmaniasis/  sandfly fever.mp. or exp sandfly fever/  Crimean-Congo haemorrhagic fever.mp. or Crimean-Congo haemorrhagic fever virus.od.  lyme disease.mp. or exp Lyme disease/  relapsing fever.mp. or exp relapsing fever/  borreliosis.mp. or Lyme disease.sh.  rickettsial diseases.mp. or exp rickettsial diseases/  (spotted fever and Q fever).mp. [mp=abstract, title, original title, broad terms, heading words, identifiers, cabicodes]  tick-borne encephalitis.mp. or tickborne diseases.sh. or Tick-borne encephalitis virus.od. or tickborne encephalitis.sh.  tularaemia.mp. or exp tularaemia/  chagas disease.mp. or Chagas' disease.sh.  American trypanosomiasis.mp. or exp Chagas' disease/  sleeping sickness.mp. or exp African trypanosomiasis/  African trypanosomiasis.mp. or exp African trypanosomiasis/  exp plague/ or plague.mp.  rickettsiosis.mp.  onchocerciasis.mp. or exp onchocerciasis/  river blindness.mp. or exp onchocerciasis/  schistosomiasis.mp. or exp schistosomiasis/  bilharziasis.mp. or exp schistosomiasis/  urban population.mp. or exp urban population/  urban health.mp. or urban areas.sh.  Cities.mp.  urban population health.mp.  exp urban environment/ or urban*.mp. or exp urban sites/  urban setting.mp.  metropolitan.mp.  municipalities.mp.  1 or 2 or 3 or 4 or 5 or 6 or 7 or 8 or 9 or 10 or 11 or 12 or 13 or 14 or 15 or 16  17 or 18 or 19 or 20 or 21 or 22 or 23 or 24  25 or 26 or 27 or 28 or 29 or 30 or 31 or 32 or 33 or 34 or 35 or 36 or 37 or 38 or 39 or 40 or 41 or 42 or 43 or 44 or 45 or 46 or 47 or 48 or 49 or 50 or 51 or 52 or 53 or 54 or 55 or 56 or 57 or 58 or 59 or 60 or 61 or 62  63 or 64 or 65 or 66 or 67 or 68 or 69 or 70  71 and 72 and 73 and 74 |

Search for relevant studies in **Scopus** Database:

| **Keywords / Descriptors** |
| --- |
| ( TITLE-ABS ( "Program Evaluation" ) OR TITLE-ABS ( "intervention evaluation" ) OR TITLE-ABS ( "Costs and Cost Analysis" ) OR TITLE-ABS ( "Evaluation stud*" ) OR TITLE-ABS ( efficiency ) OR TITLE-ABS ( "Health Program" ) OR TITLE-ABS ( "Evaluation Program" ) OR TITLE-ABS ( "Evaluation Project" ) OR TITLE-ABS ( "Project Evaluation" ) OR TITLE-ABS ( "Social Validity Research" ) OR TITLE-ABS ( "Impact analysis" ) OR TITLE-ABS ( "cost-effectiveness" ) OR TITLE-ABS ( "cost effectiveness" ) OR TITLE-ABS ( "cost-benefit" ) OR TITLE-ABS ( "cost-efficiency" ) OR TITLE-ABS ( sustainab* ) OR TITLE-ABS ( institutionalization ) OR TITLE-ABS ( routinization ) OR TITLE-ABS ( "impact assessment" ) OR TITLE-ABS ( "Impact Evaluation" ) OR TITLE-ABS ( "Program* effect*" ) OR TITLE-ABS ( " Program* outcome*" ) ) AND ( TITLE-ABS ( "Pest control" ) OR TITLE-ABS ( "Insect control" ) OR TITLE-ABS ( "Mosquito control" ) OR TITLE-ABS ( "Communicable Disease Control" ) OR TITLE-ABS ( "Tick control" ) OR TITLE-ABS ( "Infection control" ) OR TITLE-ABS ( "Biological Control Agents" ) OR TITLE-ABS ( "Integrated Vector Management" ) OR TITLE-ABS ( "Vector* control environmental management" ) OR TITLE-ABS ( "Vector Management" ) OR TITLE-ABS ( "Integrated Pest Management" ) OR TITLE-ABS ( "vector control" ) OR TITLE-ABS ( "control program*" ) OR TITLE-ABS ( "long lasting insecticidal nets" ) OR TITLE-ABS ( "larval source management" ) OR TITLE-ABS ( "indoor residual spraying" ) OR TITLE-ABS ( "chemical control" ) OR TITLE-ABS ( "biological control" ) OR TITLE-ABS ( "Habitat control" ) OR TITLE-ABS ( "Environmental control" ) ) AND ( TITLE-ABS ( "Disease Transmission" ) OR TITLE-ABS ( "Disease Vector*" ) OR TITLE-ABS ( "Communicable Disease*" ) OR TITLE-ABS ( "Neglected Disease*" ) OR TITLE-ABS ( "tropical disease*" ) OR TITLE-ABS ( "Insect Vector*" ) OR TITLE-ABS ( "Arthropod Vector*" ) OR TITLE-ABS ( "Arachnid Vector*" ) OR TITLE-ABS ( "Sandfly fever Naples virus" ) OR TITLE-ABS ( phlebovirus ) OR TITLE-ABS ( "phlebotomus fever" ) OR TITLE-ABS ( schistosomiasis ) OR TITLE-ABS ( filariasis ) OR TITLE-ABS ( "Chikungunya virus" ) OR TITLE-ABS ( "chikungunya fever" ) OR TITLE-ABS ( leishmaniasis ) OR TITLE-ABS ( "Hemorrhagic Fever Virus" ) OR TITLE-ABS ( "Crimean-Congo Hemorrhagic Fever" ) OR TITLE-ABS ( "Lyme disease*" ) OR TITLE-ABS ( "Relapsing fever" ) OR TITLE-ABS ( "Borrelia Infections" ) OR TITLE-ABS ( rickettsia ) OR TITLE-ABS ( "Rickettsia Infections" ) OR TITLE-ABS ( "Tick-Borne infections" ) OR TITLE-ABS ( "Q Fever" ) OR TITLE-ABS ( "Coxiella burneti" ) OR TITLE-ABS ( "Tick-Borne Encephalitis" ) OR TITLE-ABS ( tick-borne ) OR TITLE-ABS ( tularaemia* ) OR TITLE-ABS ( "Chagas disease" ) OR TITLE-ABS ( "African Trypanosomiasis" ) OR TITLE-ABS ( malaria ) OR TITLE-ABS ( dengue ) OR TITLE-ABS ( "Rift valley fever" ) OR TITLE-ABS ( "Rift valley fever virus" ) OR TITLE-ABS ( "Yellow fever" ) OR TITLE-ABS ( "Zika virus infection" ) OR TITLE-ABS ( "Zika virus" ) OR TITLE-ABS ( "Japanese Encephalitis" ) OR TITLE-ABS ( elephantiasis ) OR TITLE-ABS ( "West Nile fever" ) OR TITLE-ABS ( schistosomiasis ) OR TITLE-ABS ( "African Trypanosomiasis" ) OR TITLE-ABS ( plague ) OR TITLE-ABS ( "Yersinia pestis" ) OR TITLE-ABS ( rickettsia ) OR TITLE-ABS ( "Rickettsia Infections" ) OR TITLE-ABS ( onchocerciasis ) OR TITLE-ABS ( aedes ) OR TITLE-ABS ( "Dog disease*" ) OR TITLE-ABS ( "Vector-borne disease*" ) OR TITLE-ABS ( "mosquito-borne disease*" ) OR TITLE-ABS ( "mosquito disease*" ) OR TITLE-ABS ( "zoonotic disease*" ) OR TITLE-ABS ( "zoonotic pathogen*" ) OR TITLE-ABS ( "vector-borne pathogen*" ) OR TITLE-ABS ( "Emerging disease*" ) OR TITLE-ABS ( "Re-Emerging disease*" ) OR TITLE-ABS ( "Arthropod-borne virus*" ) OR TITLE-ABS ( "arthropodborne disease*" ) OR TITLE-ABS ( "arthropod-transmitted zoonose*" ) OR TITLE-ABS ( arbovirus* ) OR TITLE-ABS ( flaviviruse* ) OR TITLE-ABS ( dengue* ) OR TITLE-ABS ( zika* ) OR TITLE-ABS ( "bovine viral diarrhea virus" ) OR TITLE-ABS ( "bovine viral diarrhoea" ) OR TITLE-ABS ( alphaviruse* ) OR TITLE-ABS ( chikungunya* ) OR TITLE-ABS ( "encephalitis virus*" ) OR TITLE-ABS ( "paralytic virus*" ) OR TITLE-ABS ( trypanosome* ) OR TITLE-ABS ( "parasitic disease*" ) OR TITLE-ABS ( "bacterial disease*" ) OR TITLE-ABS ( bartonellosis ) OR TITLE-ABS ( plague* ) OR TITLE-ABS ( tularaemia* ) OR TITLE-ABS ( typhus ) OR TITLE-ABS ( "vector-borne zoonose" ) OR TITLE-ABS ( "spotted fever" ) OR TITLE-ABS ( rabie* ) ) AND ( TITLE-ABS ( "Urban Population" ) OR TITLE-ABS ( "Urban health" ) OR TITLE-ABS ( cities ) OR TITLE-ABS ( city ) OR TITLE-ABS ( urbanization ) OR TITLE-ABS ( urban* ) OR TITLE-ABS ( "urban area*" ) OR TITLE-ABS ( "urban spatial" ) OR TITLE-ABS ( "urban setting" ) OR TITLE-ABS ( "urban environment*" ) OR TITLE-ABS ( "urban ecosystem*" ) OR TITLE-ABS ( municipal* ) OR TITLE-ABS ( "Large Cities" ) OR TITLE-ABS ( "Medium Cities" ) OR TITLE-ABS ( "Small Cities" ) OR TITLE-ABS ( neighborhood* ) OR TITLE-ABS ( neighbourhood* ) OR TITLE-ABS ( town* ) OR TITLE-ABS ( slum* ) OR TITLE-ABS ( district* ) OR TITLE-ABS ( suburb* ) ) |

Search for relevant studies in **Embase** Database:

| **Keywords / Descriptors** |
| --- |
| ('evaluation study'/exp OR 'evaluation study' OR 'cost effectiveness analysis'/exp OR 'cost effectiveness analysis' OR 'evaluation of impact' OR sustainab* OR 'program evaluation'/exp OR 'program evaluation' OR 'health program'/exp OR 'health program' OR 'project evaluation' OR 'social validity research'/exp OR 'social validity research' OR 'impact analysis' OR 'cost-effectiveness'/exp OR 'cost-effectiveness' OR 'cost effectiveness'/exp OR 'cost effectiveness' OR 'cost-benefit'/exp OR 'cost-benefit' OR 'cost-efficiency' OR 'impact assessment' OR 'impact evaluation' OR 'program effectiveness'/exp OR 'program effectiveness' OR 'program outcome' OR 'costs and cost analysis'/exp OR 'costs and cost analysis' OR 'institutionalization'/exp OR institutionalization OR routinization) AND ('pest control'/exp OR 'pest control' OR 'insect control'/exp OR 'insect control' OR 'integrated pest management'/exp OR 'integrated pest management' OR 'pest management'/exp OR 'pest management' OR 'infection control'/exp OR 'infection control' OR 'biological control agent'/exp OR 'biological control agent' OR 'integrated vector management' OR 'vector ecology and management' OR 'integrated disease management approach' OR 'vector-borne diseases' OR 'vector control'/exp OR 'vector control' OR 'vector-borne disease' OR 'control programmes' OR 'control programme' OR 'vector control environmental management' OR 'vector management' OR 'integrated pest management'/exp OR 'integrated pest management' OR 'pest control'/exp OR 'pest control' OR 'mosquito control'/exp OR 'mosquito control' OR 'insect control'/exp OR 'insect control' OR 'tick control'/exp OR 'tick control' OR 'communicable disease control'/exp OR 'communicable disease control' OR 'infection control'/exp OR 'infection control' OR 'biological control agent'/exp OR 'biological control agent' OR 'long lasting insecticidal nets' OR 'larval source management' OR 'indoor residual spraying'/exp OR 'indoor residual spraying' OR 'chemical control' OR 'biological control'/exp OR 'biological control' OR 'habitat control' OR 'environmental control') AND ('disease transmission'/exp OR 'disease transmission' OR 'communicable disease'/exp OR 'communicable disease' OR 'parasite vector'/exp OR 'parasite vector' OR 'sandfly fever naples virus'/exp OR 'sandfly fever naples virus' OR 'phlebovirus'/exp OR 'phlebovirus' OR 'filariasis'/exp OR 'filariasis' OR 'chikungunya virus'/exp OR 'chikungunya virus' OR 'chikungunya'/exp OR 'chikungunya' OR 'leishmaniasis'/exp OR 'leishmaniasis' OR 'crimean-congo hemorrhagic fever virus'/exp OR 'crimean-congo hemorrhagic fever virus' OR 'lyme disease'/exp OR 'lyme disease' OR 'borrelia infection'/exp OR 'borrelia infection' OR 'rickettsia'/exp OR 'rickettsia' OR 'rickettsiaceae infection'/exp OR 'rickettsiaceae infection' OR 'q fever'/exp OR 'q fever' OR 'coxiella burnetii'/exp OR 'coxiella burnetii' OR 'tick borne encephalitis virus'/exp OR 'tick borne encephalitis virus' OR 'tularemia'/exp OR 'tularemia' OR 'chagas disease'/exp OR 'chagas disease' OR 'malaria'/exp OR 'malaria' OR 'dengue'/exp OR 'dengue' OR 'rift valley fever'/exp OR 'rift valley fever' OR 'rift valley fever virus'/exp OR 'rift valley fever virus' OR 'yellow fever'/exp OR 'yellow fever' OR 'japanese encephalitis'/exp OR 'japanese encephalitis' OR 'elephantiasis'/exp OR 'elephantiasis' OR 'west nile fever'/exp OR 'west nile fever' OR 'schistosomiasis'/exp OR 'schistosomiasis' OR 'african trypanosomiasis'/exp OR 'african trypanosomiasis' OR 'plague'/exp OR 'plague' OR 'yersinia pestis'/exp OR 'yersinia pestis' OR 'onchocerciasis'/exp OR 'onchocerciasis' OR 'aedes'/exp OR 'aedes' OR 'dog disease'/exp OR 'dog disease' OR zika OR 'mosquito'/exp OR 'mosquito' OR 'vector-borne disease' OR 'mosquito-borne disease' OR 'mosquito disease' OR 'zoonotic disease' OR 'zoonotic pathogen' OR 'vector-borne pathogen' OR 'insect vector'/exp OR 'insect vector' OR 'emerging disease' OR 're-emerging disease'/exp OR 're-emerging disease' OR 'arthropod-borne virus'/exp OR 'arthropod-borne virus' OR 'arthropodborne disease' OR 'arthropod-transmitted zoonose' OR 'neglected disease'/exp OR 'neglected disease' OR 'tropical disease'/exp OR 'tropical disease' OR 'arbovirus'/exp OR 'arbovirus' OR 'flavivirus'/exp OR 'flavivirus' OR 'bovine viral diarrhea'/exp OR 'bovine viral diarrhea' OR 'spotted fever' OR 'sandfly fever'/exp OR 'sandfly fever') AND ('urban population'/exp OR 'urban population' OR 'urban health'/exp OR 'urban health' OR 'cities'/exp OR 'cities' OR urban* OR 'urban area'/exp OR 'urban area' OR 'urban setting' OR 'urban environmental' OR 'urban spatial' OR 'urban ecosystem' OR suburb* OR municipal* OR 'big cities' OR 'large cities' OR 'medium cities' OR 'town' OR 'town'/exp OR 'small cities' OR 'urbanization'/exp OR 'urbanization' OR 'neighborhood'/exp OR 'neighborhood' OR 'neighbourhood'/exp OR 'neighbourhood' OR 'slum*') |
