# Supplementary material for: Impact, economic evaluation, and sustainability of integrated vector management in urban settings to prevent vector-borne diseases: a scoping review
Source: Infect Dis Poverty. 2018 Sep 3;7:83. doi: 10.1186/s40249-018-0464-x (PMC6120095; doi:10.1186/s40249-018-0464-x)
Supplement: Supplementary file 3 — Description of selected studies. (DOCX 66 kb) [file 40249_2018_464_MOESM3_ESM.docx]

**Additional file 2: DESCRIPTION OF SELECT STUDIES**

| Authors | Year | Title | Language | Country | Disease concerned | Type | Conclusions |
| --- | --- | --- | --- | --- | --- | --- | --- |
| 1. Espinoza-Gómez, F.; Moises Hernández-Suárez, C.; Coll-Cárdenas, R. | 2002 | Educational campaign versus malathion spraying for the control of Aedes aegypti in Colima, Mexico | English | Mexico | Dengue | Quantitative randomized controlled (trials) | An intensive EC, personalised and based on the active participation of the community leaders, is much more effective than the traditional applications of chemicals in a vertical form for the control of Aedes aegypti and the dengue transmission. |
| 1. Escudero-Támara, E.; Villareal-Amaris, G. | 2015 | Educational intervention for the control of dengue in family environments in a community in Colombia | Spanish | Colombia | Dengue | Quantitative non-randomized | The results show the effectiveness of the EI model: mobilization of schoolchildren caregivers were achieved at different stages of behavior change determine for the elimination of vector breeding sites. |
| 1. Sommerfeld, J.; Kroeger, A. | 2013 | Eco-bio-social research on dengue in Asia: A multicountry study on ecosystem and community-based approaches for the control of dengue vectors in urban and peri-urban Asia | English | Six countries: South Asia (India, Sri Lanka) and South-East Asia (Indonesia, Myanmar, Philippines, Thailand). | Dengue | Mixed Methods | Results suggests that vector management would be more sustainable when it includes: (1) involving diverse partners — including local communities, (2) targeting water container interventions that achieve a significant reduction of dengue vectors (in India, Thailand, Sri Lanka and Myanmar), and (3) utilizing novel non-insecticidal intervention tools (such as rectangular water container covers in India, sweeping nets or dragon fly nymphs in Myanmar, and copepods and screen covers for earthen jars in Thailand). |
| 1. Pai, H.-H.; Hong, Y.-J.; Hsu, E.-L. | 2006 | Impact of a short-term community-based cleanliness campaign on the sources of dengue vectors: An entomological and human behavior study | English | Taiwan | Dengue | Quantitative descriptive | The study determines that a short-term commnunity-based campaign is an effective alternative in rapidly reducing the sources of dengue vector, especially at the onset of a new epidemic. |
| 1. Samuelsen, H.; Toé, L.P.; Baldet, T.; Skovmand, O. | 2004 | Prevention of mosquito nuisance among urban populations in Burkina Faso | English | Burkina Faso | Dengue | Mixed Methods | The intervention programme, where a bio-larvicide was used to control mosquito larvae, was received positively by the local population. In the postintervention survey, they found a decrease in the level of perceived mosquito nuisance. |
| 1. Raju, A.K. | 2003 | Community mobilization in Aedes aegypti control programme by source reduction in peri-urban district of Lautoka, Viti Levu, Fiji Islands | English | Fiji Islands | Dengue | Quantitative descriptive | In this peri-urban district, refuse collection was a key requirement for the control of larval production sites. Because of water shortage problems, there is a need to design appropriate covers or biological control methods for water storage containers as an important component of a sustainable control programme. |

| Authors | Year | Title | Language | Country | Disease concerned | Type | Conclusions |
| --- | --- | --- | --- | --- | --- | --- | --- |
| 1. Vivas, E.; De Sequeda, M.G | 2003 | A game as an educational strategy for the control of Aedes aegypti in Venezuelan schoolchildren | Spanish | Venezuela | Dengue | Quantitative descriptive | As an educational tool in basic education, it has facilitated the incorporation of skills and abilities to combat dengue and its vector. In the post-implementation evaluation, students noted a considerable increase in the average score of knowledges, and the level of skills learned and deployed by students improved. " |
| 1. Caceres-Manrique, F. de M.; Angulo-Silva, M. L.; Vesga-Gomez, C. | 2010 | Efficacy of the social mobilization and the social participation in dengue control measures. | Spanish | Colombia | Dengue | Quantitative descriptive | The findings show that implementation of the strategy of social mobilization and communication adapted to the context was effective in improving knowledge, practices and "empowerment" measures of prevention and control of dengue in this community |
| 1. Wai, K.T.; Htun, P.T.; Oo, T.; Myint, H.; Lin, Z.; Kroeger, A.; Sommerfeld, J.; Petzold, M. | 2013 | Community-centred eco-bio-social approach to control dengue vectors: An intervention study from Myanmar | English | Myanmar | Dengue | Mixed Methods | Intervention effect on people’s knowledge, attitudes, and practices: Qualitative evaluations after the intervention captured that people’s awareness of appropriate vector control options for specific containers was highly improved as well as positive attitudes towards joint actions. The community-centred multi-stakeholder intervention was good in reducing vector densities (using as the PPI as the main outcome measure). In terms of sustainability and empowerment of communities, the partnership approach with targeted container interventions was found to be superior to the vertical approach. |
| 1. Toledo Romani, M.E.; Vanlerberghe, V.; Perez, D.; Lefevre, P.; Ceballos, E.; Bandera, D.; Baly Gil, A.; Van der Stuyft, P. | 2007 | Achieving sustainability of community-based dengue control in Santiago de Cuba | English | Cuba | Dengue | Mixed Methods | The creation of coordinating groups at municipal and provincial level permitted the continuous training of human resources and it contributed to strengthen the participatory processes in the communities. A community-based intervention approach promises to be sustainable. |
| 1. Neto, F.C.; Barbosa, A.A.C.; Cesarino, M.B.; Favaro, E.A.; Mondini, A.; Ferraz, A.A.; Dibo, M.R.; Vicentini, M.E. | 2006 | Dengue control in an urban area of Brazil: Impact of the Family Health Program on traditional control | Portuguese | Brazil | Dengue | Quantitative descriptive | There were significant changes concerning the increase in information about the disease and reduction in vector breeding sites. Significant increase of the proportion of people who recognized the immature forms of A. aegypti. Positive influence of training and continuing education process conducted. |

| Authors | Year | Title | Language | Country | Disease concerned | Type | Conclusions |
| --- | --- | --- | --- | --- | --- | --- | --- |
| 1. Mitchell-Fostera, K.; Ayala, E. B.; Breilh, J.; Spiegel, J.; Wilches, A. A.; Leond, T. O.; Delgado, J. A. | 2015 | Integrating participatory community mobilization processes to improve dengue prevention: an eco-bio-social scaling up of local success in Machala, Ecuador | English | Ecuador | Dengue | Quantitative randomized controlled (trials) | An integrated eco-bio-social approach to reducing PPI as a useful outcome measure of dengue transmission risk has been successful in Machala, Ecuador. |
| 1. Thalagala, N.; Tissera, H.; Palihawadana, P; Amarasinghe, A.; Ambagahawita, A.; Wilder-Smith A. et al. | 2016 | Costs of Dengue Control Activities and Hospitalizations in the Public Health Sector during an Epidemic Year in Urban Sri Lanka | English | Sri Lanka | Dengue | Quantitative descriptive | The total public sector cost of dengue control activities in Colombo district during the epidemic year of 2012 was estimated at US$971,360. About 79% of these costs were personnel costs. This was followed by consumables costs, corresponding to 16% of total costs. These results indicated a per capita cost of US$0.42 for dengue control activities in Colombo district. |
| 1. Shepard, D.S.; Halasa, Y.A.; Fonseca, D.M.; Farajollahi, A.; Healy, S.P.; Gaugler, R.; et al. | 2014 | Economic Evaluation of an Area-Wide Integrated Pest Management Program to Control the Asian Tiger Mosquito in New Jersey | English | United Stated | Several | Quantitative descriptive | Intervention years show a favorable impact of AW-IPM. The AW-IPM reduced the average number of hours lost due to mosquitoes between 2009 and 2011 by 3.30 hours per summer week; indicating a 36.4% reduction in hours lost due to mosquitoes in the intervention areas compared to control areas |
| 1. Packierisamy, P.R.; Ng, C-W.; Dahlui, M.; Venugopalan, B.; Halasa, Y.A.; Shepard, D.S. | 2015 | The Cost of Dengue Vector Control Activities in Malaysia by Different Service Providers | English | Malaysia | Dengue | Quantitative descriptive | The findings provide some evidence to rationalize delivery of dengue vector control services in Malaysia. District Health Departments spent US$5.62 million or US$679 per case and Local Authorities spent US$2.61 million or US$499 per case. The highest expenditure was for fogging, 51.0% and 45.8% of costs for District Health Departments and Local Authorities, respectively. The District Health Departments had higher resource costs for human personnel, vehicles, pesticides, and equipment. |
| 1. Orellano, P.W.; Pedroni, E. | 2008 | Análisis costo-beneficio del control de vectores en la transmisión potencial de dengue | Spanish | Argentina | Dengue | Quantitative descriptive | The total cost of the intervention strategy was higher $378,372, ie $8.05 per capita, while for the strategy of non-implementation of vector control was estimated at $106,724, or R $ 2,27 per capita. |
| 1. 17. Baly, A., González, K.; Cabrera, P.; Popa, J.C.; Toledo, M.E.; Hernández, C.; et al. | 2016 | Incremental cost of implementing residual insecticide treatment with delthametrine on top of intensive routine Aedes aegypti control | English | Cuba | Dengue | Quantitative descriptive | The annual costs of routine ACP to control Aedes aegypti in Santiago de Cuba and of three incremental RIT applications were 19.66 US$ and 3.06 US$ per household, respectively. The largest cost components for RIT were insecticide and labour expenses |

| Authors | Year | Title | Language | Country | Disease concerned | Type | Conclusions |
| --- | --- | --- | --- | --- | --- | --- | --- |
| 1. Gürtler, R.E.; Garelli, F.M.; Coto, H.D. | 2009 | Effects of a Five-Year Citywide Intervention Program To Control Aedes aegypti and Prevent Dengue Outbreaks in Northern Argentina | English | Argentina | Dengue | Quantitative non-randomized | Regression models showed that Breteau indices declined significantly compared to pre-intervention indices clustered by neighborhood, after allowing for lagged effects of temperature and rainfall, baseline Breteau index, and surveillance coverage. Larval indices seldom fell to 0 shortly after interventions at the same blocks. Large water-storage containers were the most abundant and likely to be infested. The reported incidence of dengue cases declined from 10.4 per 10,000 in 2000 to 0, and then rose to 4.5 cases per 10,000. |
| 1. Maheu-Giroux, M.; Castro, M.C. | 2014 | Cost-effectiveness of larviciding for urban malaria control in Tanzania | English | Tanzania | Malaria | Quantitative descriptive | The study has shown that, according to commonly used GDP thresholds, this intervention is very cost-effective in most transmission settings where malaria incidence is above 110–116 infections per 1,000 per year. CER for microbial larviciding were highly dependent on the assumed baseline malaria incidence rates. Using the societal perspective, net CER were estimated (in 2012 US dollars) at $43 per disability-adjusted life year averted (DALY) when malaria incidence was 902 infections per 1,000 individuals, increasing to $545 (95% UI: $337-1,558) per DALY at an incidence of 122 per 1,000. |
| 1. Vanlerberghe, V.; Villegas, E.; Oviedo, M.; Baly, A.; Lenhart, A.; McCall, P.J.; et al. | 2011 | Evaluation of the Effectiveness of Insecticide Treated Materials for Household Level Dengue Vector Control | English | Venezuela | Dengue | Quantitative randomized controlled (trials) | At distribution, the proportion of households with ≥1 ITM curtain was 79.7% in urban clusters, but decreased to 32.3%, after 18 months. The proportion of jars using ITM covers were 34.0% and 50.8% at distribution and 17.0% and 21.0% after 18 months, respectively. Prior to intervention, the BI was 8.5 in urban clusters and 42.4 in suburban clusters, and the PPI was 0.2 and 0.9, respectively. The BI showed a sustained 55% decrease. |
| 1. Baly, A.; Toledo, M.E.; Vanlerberghe, V.; Ceballos, E.; Reyes, A.; Sanchez, I.; etal. | 2009 | Cost-Effectiveness of a Community-Based Approach Intertwined with a Vertical Aedes Control Program | English | Cuba | Several | Quantitative descriptive | The average cost-effectiveness ratio for the intervention period 2001–2004, expressed as the societal cost incurred for the reduction of Aedes foci, was US$831.1 per focus in the intervention areas versus US$2,465.6 in the control areas. The intervention produced economic savings and health benefits that were sustained over the whole observation period. |
| 1. Tana, S.; Umniyati, S.; Petzold, M.; Kroeger, A.; Sommerfeld, J. | 2012 | Building and analyzing an innovative community-centered dengue-ecosystem management intervention in Yogyakarta, Indonesia | English | Indonesia | Dengue | Quantitative randomized controlled (trials) | Better community knowledge, attitude and practices in dengue prevention; increased household and community participation; improved partnership including a variety of stakeholders with prospects for sustainability; vector control efforts refocused on environmental and health issues; increased community ownership of dengue vector management including broader community development activities such as solid waste management and recycling |

| Authors | Year | Title | Language | Country | Disease concerned | Type | Conclusions | |
| --- | --- | --- | --- | --- | --- | --- | --- | --- |
| 1. Fonseca, D.M.; Unlu, I.; Crepeau, T.; Farajollahi, A.; Healy, S.P.; Bartlett-Healy, K.; Strickman, D.; Gaugler, R.; Hamilton, G.; Kline, D.; Clark, G.G. | 2013 | Area-wide management of Aedes albopictus. Part 2: Gauging the efficacy of traditional integrated pest control measures against urban container mosquitoes | English | United States | Dengue | Quantitative non-randomized | | The education campaign led to a significant reduction in adult Ae. albopictus females in 2 groups, but not in the other. The results indicate that the community of those sites responded more energetically to an outreach program, considering that the high school graduation rate in that community is 31.5% compared with 85.3% |
| 1. Kittayapong, P.; Thongyuan, S.; Olanratmanee, P.; Aumchareoun, W.; Koyadun, S.; Kittayapong, R.; Butraporn, P. | 2013 | Application of eco-friendly tools and eco-biosocial strategies to control dengue vectors in urban and peri-urban settings in Thailand | English | Thailand | Dengue | Quantitative randomized controlled (trials) | | The programme demonstrated a significant reduction in the pupae per person index during entomological surveys. The programme also raised awareness in applying eco-friendly vector control approaches and increased intersectoral and household participation in dengue control activities. |
| 1. Costa, C. M. da; Moutinho, F. F. B.; Bruno, S. F. | 2004 | The Paraty county (Rio de Janeiro, Brazil) experience in American cutaneous leishmaniasis prevention and control. | Portuguese | Brazil | Leishmaniosis | Quantitative descriptive | | There was a decrease of 31,6%, in relation to the cases reported before the intervention |
| 1. Caprara, A; Lima, J.W.O.; Peixoto, A.C.R.; Motta, C.M.V.; Nobre, J. M. S.; Sommerfeld, J.; Kroeger, A. | 2015 | Entomological impact and social participation in dengue control: a cluster randomized trial in Fortaleza, Brazil. | English | Brazil | Dengue | Quantitative randomized controlled (trials) | | Differences in terms of social participation, commitment and leadership were present in the clusters. The results showed the effectiveness of the intervention package in comparison with the routine control programme. Differences regarding the costs of the intervention were reasonable and could be adopted by public health services |
| 1. Ocampo, CB.; Gonzalez, C; Morales, CA.; Perez, M; Wesson, Dawn; Apperson, CS. | 2009 | Evaluation of community-based strategies for Aedes aegypti control inside houses. | English | Colombia | Dengue | Quantitative randomized controlled | | The lack of significant differences among the interventions, and between treated and control blocks suggested that educational activities together with periodic visits to the houses produced similar reductions of immature and adult Aedes aegypti |

| Authors | Year | Title | Language | Country | Disease concerned | Type | Conclusions |
| --- | --- | --- | --- | --- | --- | --- | --- |
| 1. Skovmand, O.; Ouedraogo, T. D. A.; Sanogo, E.; Samuelsen, H.; Toe, L. P.; Bosselmann, R.; Czajkowski, T.; Baldet, T. | 2011 | Cost of Integrated Vector Control With Improved Sanitation and Road Infrastructure Coupled With the Use of Slow-Release Bacillus sphaericus Granules in a Tropical Urban Setting | English | Burkina Faso | Malaria | Quantitative descriptive | Environmental improvements were initially costly, but demanded little subsequent expenditure. Compared with what people spent individually on mosquito prevention and malaria medicine, these measures were not expensive, but many expected the community to pay for them from existing taxes, e.g., for water treatment and disposal. It is possible to implement mosquito control with teams of young people rapidly trained to apply a biological larvicide without any tools other than an iron bar to lift cesspool lids. |
| 1. Valadez, J. J.; Devkota, B.; Pradhan, M. M.; Meherda, P.; Sonal, G. S.; Dhariwal, A.; Davis, R. | 2014 | Improving malaria treatment and prevention in India by aiding district managers to manage their programmes with local information: a trial assessing the impact of Lot Quality Assurance Sampling on programme outcomes | English | India | Malaria | Quantitative randomized controlled | Difference-in-Differences tests revealed that intervention districts exhibited significantly greater change in four of six vertical strategies (insecticide treated bed-nets and indoor residual spraying), one of six treatment seeking behaviours and four of 12 health worker capacity indicators. The control district displayed greater improvement than two intervention districts for one health worker capacity indicator. One district with poor management did not improve. |
| 1. Caldas de Castro, M.; Yamagata, Y.; Mtasiwa, D.; Tanner, M.; Utzinger, J.; Keiser, J.; et al. | 2004 | Integrated urban malaria control: a case study in Dar es Salaam, Tanzania | English | Tanzania | Malaria | Quantitative descriptive | There was a significant impact on the parasite rates immediately after the program was launched. A major contribution was the stereoscopic aerial photo interpretation routine that allowed the rapid identification of potential breeding sites and the elaboration of malaria risk maps. Qualitative assessment of costs, effectiveness, technical feasibility, and sustainability of measures allowed to prioritizate different mesures |
| 1. Chaki, P.P.; Mlacha, Y.; Msellemu, D.; Muhili, A.; Malishee, A.D.; Mtema, Z.J.; Kiware, S.S.; Zhou, Y.; Lobo, N.F.; Russell, T.L.; Dongus, S.; Govella, N.J.; Killeen, GF. | 2012 | An affordable, quality-assured community-based system for high-resolution entomological surveillance of vector mosquitoes that reflects human malaria infection risk patterns | English | 1. United Republic of Tanzania 2. UK | Malaria | Quantitative descriptive | Community Based trapping approaches could be improved with more sensitive traps, but already offer a practical, safe and affordable system for routine programmatic mosquito surveillance and clusters could be distributed across entire countries by adapting the sample submission and quality assurance procedures accordingly. |
| 1. Ordoñez-González, J.G.; Thirion, J.; García- Orozco, A.; Rodríguez, A.D. | 2011 | Effectiveness of indoor ultra-low volume application of aqua RESLINH (R) super during an emergency | English | Mexico | Dengue | Quantitative descriptive | Aqua ReslinH Super showed effectiveness against adult mosquitoes Aedes aegypti. After the spraying, ovitrap data showed no mosquito adults present 4 days after the applications, and only 1 ovitrap out of 60 positive 8 days after the intervention. |

| Authors | Year | Title | Language | Country | Disease concerned | Type | Conclusions |
| --- | --- | --- | --- | --- | --- | --- | --- |
| 1. Pacheco-Coral, A.P.; Quiñones-Pinzón, M.L.; Serrato-Pomar, I.M.; Rivas-Muñoz, F.A. | 2010 | Evaluación preliminar de la Estrategia de Información, Comunicación y Educación para el control del Aedes aegypti, en La Dorada, Colombia | Spanish | Colombia | Dengue | Quantitative descriptive | The strategy was known by 80%, the presence of women as head of household was associated with the absence of larvae. Washing containers at least once a week was effective in the presence of immature. |
| 1. Betancourt Betancourt, J.A. ; García Rodríguez, C.J. ; Alfonso, P.J.; Llambias Peláez, J.J. ; García Fariñas, A. | 2011 | Análisis de eficiencia relativa en el control del Aedes aegypti del municipio Camagüey | Spanish | Cuba | Dengue | Quantitative descriptive | For the study outcome, possible management improvements seen in some areas. By properly using the resources at their disposal: using strictly necessary and competent professional staff, adequate use of insecticides and other resources. The possibility of saving some of the resources used in the spraying is possible in all areas. |
| 1. Basso, C.; García da Rosa, E.; Romero, S.; González, C.; Lairihoy, R.; Roche, I.; Caffera, R.M.; da Rosa, R., Calfani, M.; Alfonso-Sierra, E.; Petzold, M.; Kroeger, A.; Sommerfeldh, J. | 2014 | Improved dengue fever prevention through innovative intervention methods in the city of Salto, Uruguay | English | Uruguay | Dengue | Quantitative randomized controlled (trials) | The vector densities in intervention clusters on average increased less than those in the control clusters |
| 1. Quintero, J.; García-Betancourt, T.; Cortés, S.; García, D.; Alcala, L.; González-Uribe, C.; Brochero, H.; Carrasquilla, G. | 2014 | Effectiveness and feasibility of long-lasting insecticide-treated curtains and water container covers for dengue vector control in Colombia: a cluster randomised trial | English | Colombia | Dengue | Quantitative randomized controlled (trials) | The Breteau Index fell from 14 to 6 in the intervention group and from 8 to 5 in the control group. In the intervention group, the PPI index showed a clear decline of 71% compared with 25% in the control group. The results obtained in our study indicate that the intervention package can reduce dengue vector density. |
| 1. Ogoma, S.B.; Kannady K.; Sikulu M.; Chaki, P.P.; Govella N.J.; Mukabana W.R.; Killeen G.F. | 2009 | Window screening, ceilings and closed eaves as sustainable Window screening, ceilings and closed eaves as sustainable ways to control malaria in Dar es Salaam, Tanzania | English | Tanzania | Malaria | Quantitative descriptive | The IC strategy on dengue transmission was known by 80%, the presence of women as head of household was associated with the absence of larvae. Washing tank at least once a week was effective in the presence of immature. |
| 1. Maheu-Giroux, M.; Castro, M.C. | 2013 | Do malaria vector control measures impact disease-related behaviour and knowledge? Evidence from a large-scale larviciding intervention in Tanzania | English | Tanzania | Malaria | Quantitative descriptive | The impact of the larviciding intervention on knowledge of malaria symptoms was also shown to be statistically significant |

| Authors | Year | Title | Language | Country | Disease concerned | Type | Conclusions |
| --- | --- | --- | --- | --- | --- | --- | --- |
| 1. Vanlerberghe, V.; Toledo, M.E.; Rodríguez, M.; Gomez, D.; Baly, A.; Benitez, J.R; Van der Stuyft, P. | 2009 | Community involvement in dengue vector control: cluster randomised trial | English | Cuba | Dengue | Quantitative randomized controlled (trials) | All clusters received the intended intervention; they completed the study protocol up to February 2006 and were included in the analysis. At last, infestation levels in the intervention clusters were significantly lower than those in the control clusters |
| 1. Geissbühler, Y.; Kannad, K.; Chaki, P.P.; Emidi, B.; Govella, N.J.; Mayagaya, V.; Kiama, M.; Mtasiwa, D.; Mshinda, H.; Lindsay, S.W.; Tanner, M.; Fillinger, U.; de Castro, M.C.; Killeen, G.F. | 2009 | Microbial Larvicide Application by a Large-Scale, Community-Based Program Reduces Malaria Infection Prevalence in Urban Dar Es Salaam, Tanzania | English | Tanzania | Malaria | Quantitative randomized controlled (trials) | Larviciding reduced malaria prevalence and complemented existing protection provided by insecticide-treated nets. Larviciding may represent a useful option for integrated vector management in Africa, particularly in its rapidly growing urban centres. |
| 1. Ocampo, C.B.; Mina, N.J.; Carabalí, M.; Alexander, N.; Osorio, L. | 2014 | Reduction in dengue cases observed during mass control of Aedes (Stegomyia) in street catch basins in an endemic urban area in Colombia | English | Colombia | Dengue | Quantitative randomized controlled (trials) | A significant decrease in catch basins positivity for Aedes larvae was observed after each monthly treatment (p < 0.001). Over the intervention period, a reduction in the dengue incidence in Buga was observed (rate ratio 0.19, 95% CI 0.12–0.30, p < 0.0001) after adjusting for autocorrelation and controlling with a neighboring town, Palmira |
| 1. Noazin, S.; Shirzadib, M.R.; Kermanizadeh, A.; Yaghoob-Ershadie, M.R.; Sharifi, I. | 2013 | Effect of large-scale installation of deltamethrin-impregnated screens and curtains in Bam, a major focus of anthroponotic cutaneous leishmaniasis in Iran | English | Iran | Leishmaniasis | Quantitative randomized controlled (trials) | Findings indicate the short-term effectiveness of such preventive measures but highlight the necessity of long-term, sustainable strategies. Results also suggest that shrinkage in the human reservoir pool in response to the intervention may play a significant role in prolongation of the preventive effect beyond the insecticidal life of impregnated materials in foci of anthroponotic cutaneous leishmaniasis and possibly anthroponotic visceral leishmaniasis due to L. donovani. |
