# Supplementary material for: Impact, economic evaluation, and sustainability of integrated vector management in urban settings to prevent vector-borne diseases: a scoping review
Source: Infect Dis Poverty. 2018 Sep 3;7:83. doi: 10.1186/s40249-018-0464-x (PMC6120095; doi:10.1186/s40249-018-0464-x)
Supplement: Supplementary file 4 — Description of outcome measures for impact. (DOCX 77 kb) [file 40249_2018_464_MOESM4_ESM.docx]

**Additional file 3: DESCRIPTION OF OUTCOME MEASURES FOR IMPACT**

| **Outcomes measures** | **Description** |
| --- | --- |
| **USE OF MEASURES FOR VECTOR CONTROL** | |
| Insecticide treated bed-nets | Proportion of people protected and proportion of children protected (1) |
| Indoor residual spraying | Proportion of people protected and proportion of children protected (1) |
| Insecticide treated materials (ITM) curtain coverage | Proportion of households with ≥1 ITM curtain (2) |
| **CONTROL VECTOR** | |
| **Intermediate outcomes measures** | |
| Reduction of breeding places | - Number of positive containers with larva and/or pupae by house (C+/H) (3) (4) (5) - Global average of the positive containers with larva and/or pupae (C+/C) (3) - Percentage of water-filled containers positive for larvae and/or pupae (6–8) - Catch basins positive for larvae and pupae (9) |
| **Final outcomes measures** | |
| **Dengue** | |
| Pupa per person index -PPI- | Number of Pupa by person (2,7,8,10–15) |
| Pupal Index (PI) | Mean number of pupae per house (8) (5) |
| Pupae per hectare index (PHI) | Number of pupae per hectare index (PHI) (8) |
| Breteau index | Number of mosquito positive containers per house (2,8,7,15,16,6) |
| Premises or house Index | Percentage of houses positive for larvae (8,7,15,6) |
| Density adult Aedes aegypti | - Adult index: percentage of houses infested with adult mosquitoes (17) - Density immature Aedes aegypti: percentage of houses infested with adult mosquitoes (17) - Extensiveness of the distribution of Aedes mosquitoes in a particular area surveyed (17,18) |
| Ovitrap index | - Number of traps with laid eggs/total number of traps (19) - Mean egg collection per ovitrap, corrected (so all counts are equivalent to 7 days of collection) (20) |
| **Malaria** | |
| Parasite rates | Annual parasite rates (21) |
| Entomological inoculation rate | Vector mosquito densities combined with subsequent laboratory analysis of captured specimens for sporozoite-stage parasite infection prevalence (22) |
| **HEALTH MEASURES** | |
| Incidence of illness | Number of new cases per population at risk in the period (16) (9), (22) (23,24). |
| **SOCIAL MEASURES** | |
| Knowledge | Knowledge and beliefs symptoms and transmission and control measures (13) (25–29) |
| **Change behaviour, abilities and capacities** | Change behavior on population to control the breeding places (4,6,19)  Abilities and adequate practices for controlling the vector (25)  Health worker capacity indicators (1) |
| **Empowerment and participation** | Empowerment of prevention and control measures (11,13,26)  Household participation in vector control activities (30)  Intersectoral participation in vector control activities (30) |

**References**

1. Valadez JJ, Devkota B, Pradhan MM, Meherda P, Sonal GS, Dhariwal A, et al. Improving malaria treatment and prevention in India by aiding district managers to manage their programmes with local information: a trial assessing the impact of Lot Quality Assurance Sampling on programme outcomes. Trop Med Int Health. 2014 Oct;19(10):1226–36.

2. Vanlerberghe V, Villegas E, Oviedo M, Baly A, Lenhart A, McCall PJ, et al. Evaluation of the effectiveness of insecticide treated materials for household level dengue vector control. PLoS Negl Trop Dis. 2011 Mar;5(3):e994.

3. Espinoza-Gomez F, Hernandez-Suarez CM, Coll-Cardenas R. Educational campaign versus malathion spraying for the control of Aedes aegypti in Colima, Mexico. J Epidemiol Community Health. 2002 Feb;56(2):148–52.

4. Escudero-Tamara E, Villareal-Amaris G. [Educational intervention for the control of dengue in family environments in a community in Colombia]. Rev Peru Med Exp Salud Publica. 2015;32(1):19–25.

5. Pacheco-Coral A del P, Quinones-Pinzon ML, Serrato-Pomar IM, Rivas-Munoz FA. [Evaluating an Information, Education and Communication (IEC) strategy which was adopted for Aedes aegypti control in La Dorada, Colombia]. Rev Salud Publica (Bogota). 2010 Jun;12(3):380–90.

6. Raju A. Community Mobilization in Aedes aegypti Control Programme by Source Reduction in Peri-Urban District of Lautoka, Viti Levu, Fiji Islands. Dengue Bull. 2003;27:149–55.

7. Caprara A, Lima JWDO, Peixoto ACR, Motta CMV, Nobre JMS, Sommerfeld J, et al. Entomological impact and social participation in dengue control: a cluster randomized trial in Fortaleza, Brazil. Trans R Soc Trop Med Hyg. 2015 Feb;109(2):99–105.

8. Basso C, Garcia da Rosa E, Romero S, Gonzalez C, Lairihoy R, Roche I, et al. Improved dengue fever prevention through innovative intervention methods in the city of Salto, Uruguay. Trans R Soc Trop Med Hyg. 2015 Feb;109(2):134–42.

9. Ocampo CB, Mina NJ, Carabali M, Alexander N, Osorio L. Reduction in dengue cases observed during mass control of Aedes (Stegomyia) in street catch basins in an endemic urban area in Colombia. Acta Trop. 2014 Apr;132:15–22.

10. Sommerfeld J, Kroeger A. Eco-bio-social research on dengue in Asia: a multicountry study on ecosystem and community-based approaches for the control of dengue vectors in urban and peri-urban Asia. Pathog Glob Health. 2012 Dec;106(8):428–35.

11. Wai KT, Htun PT, Oo T, Myint H, Lin Z, Kroeger A, et al. Community-centred eco-bio-social approach to control dengue vectors: an intervention study from Myanmar. Pathog Glob Health. 2012 Dec;106(8):461–8.

12. Mitchell-Foster K, Ayala EB, Breilh J, Spiegel J, Wilches AA, Leon TO, et al. Integrating participatory community mobilization processes to improve dengue prevention: an eco-bio-social scaling up of local success in Machala, Ecuador. Trans R Soc Trop Med Hyg. 2015 Feb;109(2):126–33.

13. Tana S, Umniyati S, Petzold M, Kroeger A, Sommerfeld J. Building and analyzing an innovative community-centered dengue-ecosystem management intervention in Yogyakarta, Indonesia. Pathog Glob Health. 2012 Dec;106(8):469–78.

14. Quintero J, Garcia-Betancourt T, Cortes S, Garcia D, Alcala L, Gonzalez-Uribe C, et al. Effectiveness and feasibility of long-lasting insecticide-treated curtains and water container covers for dengue vector control in Colombia: a cluster randomised trial. Trans R Soc Trop Med Hyg. 2015 Feb;109(2):116–25.

15. Vanlerberghe V, Toledo ME, Rodriguez M, Gomez D, Baly A, Benitez JR, et al. Community involvement in dengue vector control: cluster randomised trial. BMJ. 2009 Jun;338:b1959.

16. Thalagala N, Tissera H, Palihawadana P, Amarasinghe A, Ambagahawita A, Wilder-Smith A, et al. Costs of Dengue Control Activities and Hospitalizations in the Public Health Sector during an Epidemic Year in Urban Sri Lanka. PLoS Negl Trop Dis. 2016 Feb;10(2):e0004466.

17. Ocampo CB, Gonzalez C, Morales CA, Perez M, Wesson D, Apperson CS. Evaluation of community-based strategies for Aedes aegypti control inside houses. Biomedica. 2009 Jun;29(2):282–97.

18. Ordonez Gonzalez JG, Thirion J, Garcia Orozco A, Rodriguez AD. Effectiveness of indoor ultra-low volume application of Aqua Reslin(R) Super during an emergency. J Am Mosq Control Assoc. 2011 Jun;27(2):162–4.

19. Pai H-H, Hong Y-J, Hsu E-L. Impact of a short-term community-based cleanliness campaign on the sources of dengue vectors: an entomological and human behavior study. J Environ Health. 2006;68(6):35–9.

20. Fonseca DM, Unlu I, Crepeau T, Farajollahi A, Healy SP, Bartlett-Healy K, et al. Area-wide management of Aedes albopictus. Part 2: gauging the efficacy of traditional integrated pest control measures against urban container mosquitoes. Pest Manag Sci. 2013 Dec;69(12):1351–61.

21. Caldas de Castro M, Yamagata Y, Mtasiwa D, Tanner M, Utzinger J, Keiser J, et al. Integrated urban malaria control: a case study in dar es salaam, Tanzania. Am J Trop Med Hyg. 2004 Aug;71(2 Suppl):103–17.

22. Geissbuhler Y, Kannady K, Chaki PP, Emidi B, Govella NJ, Mayagaya V, et al. Microbial larvicide application by a large-scale, community-based program reduces malaria infection prevalence in urban Dar es Salaam, Tanzania. PLoS One. 2009;4(3):e5107.

23. DA COSTA CM, MOUTINHO FFB, BRUNO SF. A experiência do município de Paraty (Rio de Janeiro, Brasil) na prevenção e controle da leishmaniose tegumentar americana. Parasitol Latinoam [Internet]. 2004 Jul [cited 2016 Oct 27];59(3–4):110–4. Available from: http://www.scielo.cl/scielo.php?script=sci_arttext&pid=S0717-77122004000300004&lng=es&nrm=iso&tlng=es

24. Noazin S, Shirzadi MR, Kermanizadeh A, Yaghoobi-Ershadi M-R, Sharifi I. Effect of large-scale installation of deltamethrin-impregnated screens and curtains in Bam, a major focus of anthroponotic cutaneous leishmaniasis in Iran. Trans R Soc Trop Med Hyg. 2013 Jul;107(7):444–50.

25. Vivas E, Guevara De Sequeda M. [A game as an educational strategy for the control of Aedes aegypti in Venezuelan schoolchildren]. Rev Panam Salud Publica. 2003 Dec;14(6):394–401.

26. Caceres-Manrique F de M, Angulo-Silva ML, Vesga-Gomez C. [Efficacy of the social mobilization and the social participation in dengue control measures]. Biomedica. 2010;30(4):539–50.

27. Chiaravalloti Neto F, Barbosa AAC, Cesarino MB, Favaro EA, Mondini A, Ferraz AA, et al. [Dengue control in an urban area of Brazil: impact of the Family Health Program on traditional control]. Cad Saude Publica. 2006 May;22(5):987–97.

28. Maheu-Giroux M, Castro MC. Cost-effectiveness of larviciding for urban malaria control in Tanzania. Malar J. 2014;13:477.

29. Maheu-Giroux M, Castro MC. Do malaria vector control measures impact disease-related behaviour and knowledge? Evidence from a large-scale larviciding intervention in Tanzania. Malar J. 2013 Nov;12:422.

30. Kittayapong P, Thongyuan S, Olanratmanee P, Aumchareoun W, Koyadun S, Kittayapong R, et al. Application of eco-friendly tools and eco-bio-social strategies to control dengue vectors in urban and peri-urban settings in Thailand. Pathog Glob Health. 2012 Dec;106(8):446–54.
